# Raman enhancement on ultra-clean graphene quantum dots produced by quasi-equilibrium plasma-enhanced chemical vapor deposition

**DOI:** 10.1038/s41467-017-02627-5

**Published:** 2018-01-15

**Authors:** Donghua Liu, Xiaosong Chen, Yibin Hu, Tai Sun, Zhibo Song, Yujie Zheng, Yongbin Cao, Zhi Cai, Min Cao, Lan Peng, Yuli Huang, Lei Du, Wuli Yang, Gang Chen, Dapeng Wei, Andrew Thye Shen Wee, Dacheng Wei

**Affiliations:** 1 State Key Laboratory of Molecular Engineering of Polymers, Fudan University, Shanghai, 200433 China; 2 Department of Macromolecular Science, Fudan University, Shanghai, 200433 China; 3 Shanghai National Laboratory of Infrared Physics and Institute of Technical Physics, Chinese Academy of Science, Shanghai, 200433 China; 4 Chongqing Key Laboratory of Multi-scale Manufacturing Technology, Chongqing Institute of Green and Intelligent Technology, Chinese Academy of Sciences, Chongqing, 400714 China; 5 Department of Physics, National University of Singapore, 2 Science Drive 3, Singapore, 117542 Singapore

## Abstract

Graphene is regarded as a potential surface-enhanced Raman spectroscopy (SERS) substrate. However, the application of graphene quantum dots (GQDs) has had limited success due to material quality. Here, we develop a quasi-equilibrium plasma-enhanced chemical vapor deposition method to produce high-quality ultra-clean GQDs with sizes down to 2 nm directly on SiO_2_/Si, which are used as SERS substrates. The enhancement factor, which depends on the GQD size, is higher than conventional graphene sheets with sensitivity down to 1 × 10^−9^ mol L^−1^ rhodamine. This is attributed to the high-quality GQDs with atomically clean surfaces and large number of edges, as well as the enhanced charge transfer between molecules and GQDs with appropriate diameters due to the existence of Van Hove singularities in the electronic density of states. This work demonstrates a sensitive SERS substrate, and is valuable for applications of GQDs in graphene-based photonics and optoelectronics.

## Abstract (web summary)

Surface-enhanced Raman spectroscopy (SERS) is a promising technology for sensitive optical sensors, generally using rough metal films. Here, Liu et al. synthesize high-quality graphene quantum dot films which offer a large SERS enhancement due to a strong light-matter interaction with Van Hove singularities.

## Introduction

Surface-enhanced Raman spectroscopy (SERS) is a promising analytical technique which enables single molecule sensitive detection and with chemical specificity^1,2^. Since its discovery by Fleischman et al. in 1974^3^, researchers have made great efforts to find an ideal SERS-active substrate with higher sensitivity, better uniformity and reproducibility. Until now, the most commonly used SERS substrates are noble metals such as silver, gold and copper with rough surfaces. However, cost, stability, biological compatibility and environmental considerations have motivated the search for new SERS substrates^4,5^. Compared with noble metals, graphene has a one-atomic-thick uniform structure and has advantages such as low cost, biocompatibility, chemical stability, and high adsorptivity. Meanwhile, the enhancement effect of graphene can be further amplified by combining it with metal substrates^6^. All of above make graphene one of the potential substrates for future SERS applications^7^. Until now, not only exfoliated graphene, but also graphene oxide and hydrogen-terminated graphene have shown much promise^8–11^. However, only the chemical effect contributes to the enhancement, and the graphene has high visible light transmission^12^. Therefore, only a 2-fold to 17-fold enhancement of the Raman signal (phthalocyanine) has been observed on graphene compared to that on an SiO_2_/Si substrate, which is much lower than demonstrated with noblemetal substrates^8^.

Due to quantum confinement and edge effects, graphene quantum dots (GQDs), nanometer-sized fragments of graphene, exhibit unique properties such as photoluminescence and slow hot-carrier relaxation^13–15^. Compared with conventional graphene sheets, GQDs have larger specific surface areas and more accessible edges, which results in a more effective adsorption of target molecules^12^. This, along with its unique photoelectrical properties, probably leads to stronger SERS signals for detecting target molecules in solution^12,16,17^. However, SERS applications have had limited success due to the poor quality of the GQDs produced by existing solution processes such as the hydro-/solvo-thermal route and solution chemistry^13–15^. The functional groups, defects and especially the impurities on the GQDs reduce the charge transfer between GQDs and target molecules, inducing a dramatic decrease of the SERS signals down to a level lower than that on conventional graphene sheets^12^.

Here, we find that in a quasi-equilibrium state of graphene etching and nucleation in plasma, GQDs with different sizes are controllably grown on SiO_2_/Si without metal catalysts. Based on this finding, we develop a solution-free method called quasi-equilibrium plasma-enhanced chemical vapor deposition (qe-PECVD) to produce high-quality ultra-clean GQDs directly on SiO_2_/Si. The PECVD GQDs (P-GQDs) are highly crystalline, atomically clean with a low defect density and can be directly used as SERS substrates without the need for any post-growth transfer processes. Raman measurements reveal a size-dependent enhancement behavior with the P-GQDs. With appropriate diameters, high enhancement efficiency is achieved with a factor comparable or much higher than that on conventional graphene sheets. In practical applications, Raman signal of rhodamine 6G (R6G) in ethanol solution is detectable even at a concentration as low as 10^−9^ mol L^−1^, lower than that on mechanically exfoliated graphene (10^−8^ mol L^−1^)^8^. The ab initio density functional theory (DFT) calculation attributes the mechanism of the high sensitivity partly to the enhanced charge transfer, which arises from strong light-matter interactions due to the existence of Van Hove singularities (VHS) in the density of states (DOS) of the P-GQDs, and the matched energy alignment between the molecule and the P-GQDs with appropriate diameters.

## Results

### Growth of P-GQDs by qe-PECVD

**Fig. 1:**
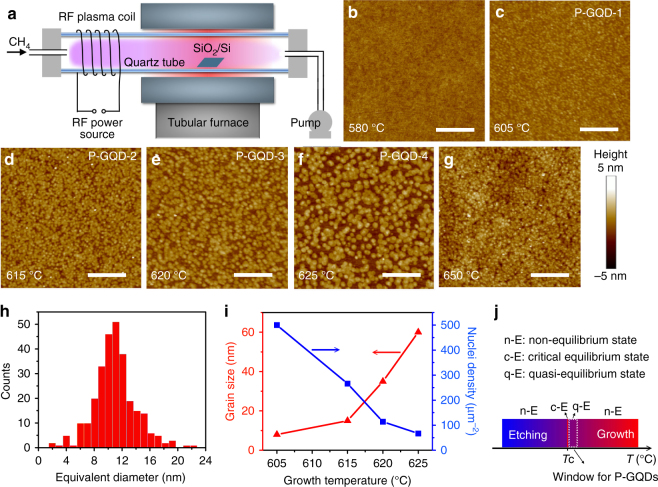
Growth of P-GQDs by qe-PECVD. **a** Schematic illustrations of the plasma-enhanced chemical vapor deposition (PECVD) system. **b**–**g** Atomic force microscope (AFM) images of samples grown at 580 °C, 605 °C, 615 °C, 620 °C, 625 °C and 650 °C, respectively. **h** The diameter distribution of the P-GQD-2 sample. **i** Relationship between grain size, nuclei density and temperature. **j** Quasi-equilibrium, critical equilibrium and non-equilibrium states at different temperatures. The scale bars in **b**–**g** are 500 nm

The PECVD system is illustrated in Fig. 1a. A cleaned SiO_2_ (300 nm thick)/Si substrate was placed in the center of the horizontal quartz tube mounted inside a tubular furnace. Methane (CH_4_, 5 sccm, 120 mTorr) plasma was generated upstream by a remote radiofrequency plasma generator (13.56 MHz, 20 W). P-GQDs were obtained on the SiO_2_/Si after 10 min growth at 580 °C, 605 °C (P-GQD-1), 615 °C (P-GQD-2), 620 °C (P-GQD-3), 625 °C (P-GQD-4) and 650 °C. Optical and scanning electron microscopy (SEM) images of the as-grown P-GQDs (Supplementary Fig. 1) show that the samples are clean without visible impurities on the whole surface of the SiO_2_/Si. Atomic force microscopy (AFM) images (Fig. 1b–g) show that no graphene is obtained after growth at 580 °C, while a thick film with random carbon clusters covers the whole SiO_2_/Si surface after growth at 650 °C. The growth of GQDs only takes place in a temperature window of 605–625 °C. The size of P-GQDs enlarges with increasing growth temperature, and the corresponding size range measured by AFM is about 3–6, 6–15, 25–50, and 45–80 nm for P-GQD-1–4, respectively (Fig. 1h, i). The sizes measured by AFM are slightly larger than the physical size of the P-GQDs due to the finite radius of the AFM tip. The thickness ranges from 0.7 to 1.2 nm excluding the roughness of SiO_2_/Si, equivalent to ~1–2 layer GQDs.

The results are in a good agreement with our previous work^18^, which reveals that there is a competition between the effects of graphene etching and growth in PECVD, and efficient catalyst-free growth of graphene crystals only takes place in an equilibrium state or steady state between these two competition processes at a critical temperature (*T*_c_) (Supplementary Fig. 2–4, Supplementary Note 1). In our experiments (Fig. 1j), *T*_c_ is around 600 °C. As a result, in a non-equilibrium state at 580 °C or 650 °C, the etching effect from hydrogen radicals or the nucleation and growth effect from carbon or hydrocarbon radicals dominates^19–21^, resulting in non-growth or formation of a carbon cluster film on the SiO_2_/Si, respectively. In a critical equilibrium state at *T*_c_, the growth of hexagonal graphene crystals (Supplementary Fig. 2) is expected, as demonstrated previously^18^. Here P-GQDs are produced at a temperature slightly higher than *T*_c_. Thus, in a quasi-equilibrium state, both the nucleation of GQDs and the growth of the crystal size are realized in a controllable manner. The domain size and nuclei density verses temperature are illustrated in Fig. 1i, showing that the domain size increases and the nuclei density decreases with increasing growth temperature.

### Detailed characterization of the P-GQDs

**Fig. 2:**
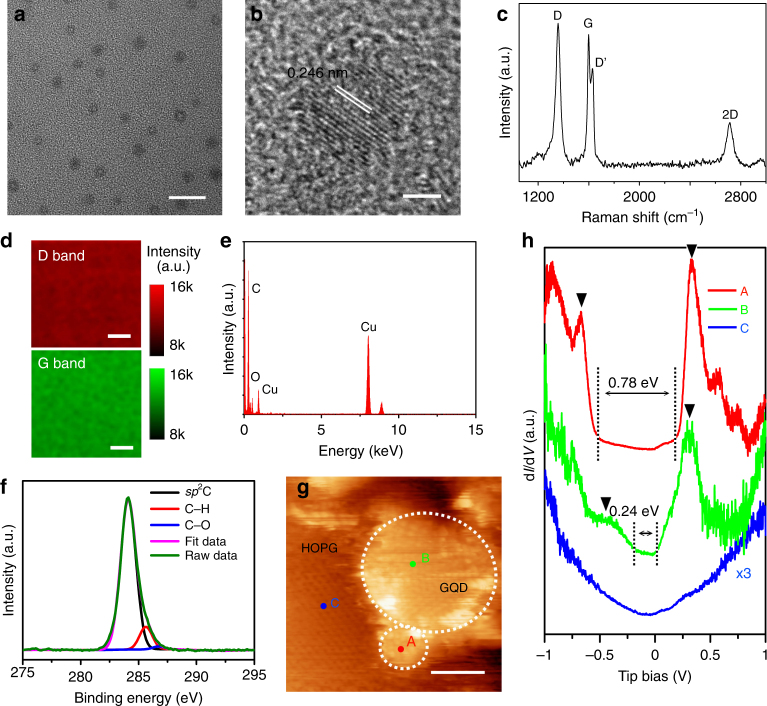
Characterization of the P-GQDs. **a** High-resolution transmission electron microscope (HRTEM) image and **b** an enlarged image and **c** Raman spectrum of the graphene quantum dots produced by PECVD (P-GQDs). **d** Raman mapping of the integrated intensity of the D band and the G band of a P-GQD sample. **e** X-ray energy dispersive spectrometer (EDS) spectrum, **f** X-ray photoelectron spectroscopy (XPS) C1s spectrum of the P-GQDs. **g** Scanning tunnelling microscopy (STM) image and **h** scanning tunnelling spectroscopy (STS) spectra of P-GQDs grown on highly oriented pyrolytic graphite (HOPG). The d*I*/d*V* curves are collected from the points indicated by colored dots in **g**. The arrows in **h** indicate the positions of Van Hove singularities (VHS). The scale bars are 20 nm in **a**, 2 nm in **b**, 10 μm in **d** and 2 nm in **g**

High resolution transmission electron microscope (HRTEM) images (Fig. 2a, Supplementary Fig. 5) show the P-GQDs have sizes of about 3–8 nm. In the enlarged images (Fig. 2b, Supplementary Figs. 6 and 7), despite the non-flat and amorphous nature of carbon membrane on the TEM grid, the (1120) crystal lattice of graphene (~ 0.246 nm) is resolved, indicating the high crystalline nature of the sample^22^. In the Raman spectra (Fig. 2c, Supplementary Fig. 8), obvious D and D′ bands imply that large amount of edge structures exist as a result of the small size of P-GQDs^18^. Homogeneous intensity of the D band and the G band in Raman mapping (Fig. 2d) reveals the high uniformity of the P-GQDs over a large area. The X-ray energy dispersive spectrometer (EDS) spectrum (Fig. 2e) exhibits a strong C element peak without any other peaks except copper and oxygen (from CuO_*x*_ and absorbed O_2_ or H_2_O on the copper TEM grid), indicating the high purity of the sample. The high purity is also supported by the wide survey X-ray photoelectron spectroscopy (XPS) spectrum (Supplementary Fig. 9), which shows the predominant presence of C. The XPS C1s spectrum (Fig. 2f) has two peaks which can be assigned as *sp*^2^ C (284.4 eV) and C–H (~285.3 eV). The dominant peak at 284.4 eV confirms that most of the C atoms are arranged in a conjugated honeycomb lattice, while the C–H peak is attributed to large amount of the H-terminated edges. Moreover, a tiny peak at about 286.7 eV is originated from the adsorbed O or background noise, consistent with the result reported previously^20^. We also measured the XPS spectra of the GQDs produced via solution processes (S-GQDs). XPS C1s spectrum (Supplementary Fig. 10) of the S-GQDs has large shoulder peaks at 286.4 eV and 288.1 eV, corresponding to C–O and C=O bonds, respectively. The absence of obvious C–O and C=O peaks, which usually appear in the S-GQDs^23^, implies that the P-GQDs have high quality without CO_*x*_ functional groups.

Moreover, we measured the photoluminescence (PL) spectrum of the P-GQDs. Although the PL mechanism still remains controversial, previous research suggests that the intrinsic quantum confinement effect might not be the main mechanism of the PL^24^. Instead, extrinsic factors such as functional groups, heteroatom doping and defects play extremely important role in achieving strong PL^25–28^. P-GQDs are perfectly crystallized without doping or functional groups and, as a result, the quantum confinement effect should be the only available origin of the PL. Thus, compared with S-GQDs, the PL signal of the P-GQD samples was too weak to be measured, probably owing to the high sample quality as well as the small number of P-GQDs in the beam spot.

To clarify the atomic and electrical structure, we performed low temperature scanning tunnelling microscopy (STM) studies on the P-GQDs. Highly oriented pyrolytic graphite (HOPG) was used as the growth substrate in qe-PECVD, which provided an inert conductive surface for both growth and STM analysis. The representative STM images (Fig. 2g, Supplementary Fig. 11) show that the P-GQDs are mono-layered with a well-defined crystalline structure. Both the surface and edges are atomically clean without impurities. As a result of the quasi-equilibrium growth process, low density defects are observed. Previous research shows that perfect hexagonal graphene crystals (Supplementary Fig. 2) can only be prepared in a critical equilibrium growth process at *T*_c_^18^, thus at a temperature closer to *T*_c_, P-GQDs with lower defect density and better shape and size distribution are expected. Due to the quantum confinement effect, significant changes in the local DOS of the P-GQDs are identified by scanning tunnelling spectroscopy (STS, Fig. 2h). On the HOPG, a typical d*I*/d*V* of graphite is recorded with no band gap, and the depression of the DOS occurs near the Dirac point (0 eV)^29^. However, in the d*I*/d*V* recorded on the P-GQDs, a series of sharp peaks appear which correspond to VHS as a result of quantum confinement in a zero-dimensional structure^30,31^. Near the Fermi level, an obvious band gap exists, indicating the semiconducting nature of the GQDs. Previous theoretical studies reveal that the band structure of the quantum dots strongly depends on the size and shape^32^. The band gaps of the 2 nm P-GQD and 6 nm P-GQD are about 0.78 eV and 0.24 eV, respectively, in good agreement with the energy level spacing (∆*E*) calculated by Equation 1^32,33^,

1 $$\Delta E = h\,\nu _{\mathrm{F}}/2\,L \approx 1.67\,{\mathrm{eV}}/L$$

where *h* is Planck’s constant, *L* is the size of GQDs generally given in nm, and *ν*_F_ = 8.1 × 10^5^ m s^−1^ is the Fermi velocity for graphene. According to equation 1, the band gap of our samples is expected in the range of 28 meV to 800 meV. This value is greater than that of the thermal fluctuations at the room temperature (26 meV), thus the samples behave like quantum dots rather than conventional graphene sheets.

### Surface Raman enhancement on P-GQDs

**Fig. 3:**
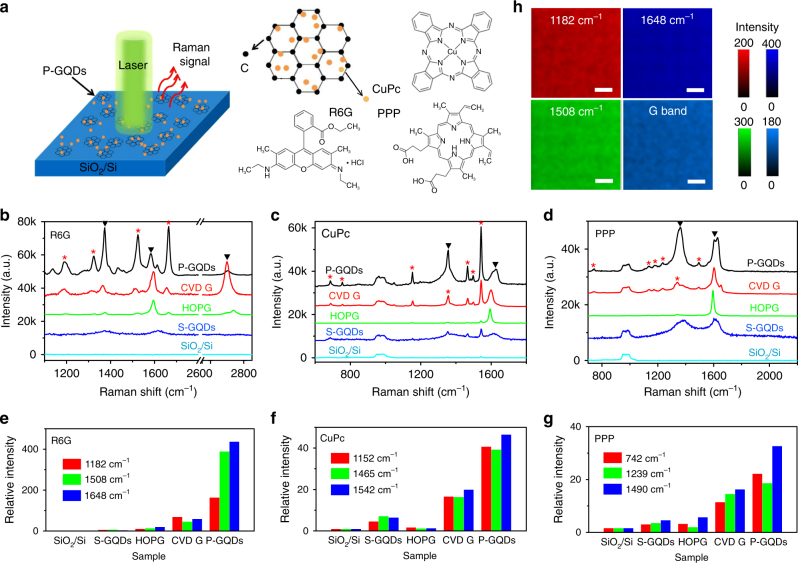
Raman enhancement of thermally evaporated R6G, CuPc, PPP on P-GQDs. **a** Schematic of a P-GQD substrate for Raman measurement. **b**–**d** Raman spectra of thermally evaporated rhodamine 6G (R6G), copper phthalocyanine (CuPc) and Protopphyrin IX (PPP) on SiO_2_/Si, graphene quantum dots produced by solution processes (S-GQDs), HOPG, transferred chemical vapor depostion (CVD) graphene and P-GQDs, respectively. Black arrows indicate the peaks from graphene, and red stars indicate the peaks from the target molecule. **e**–**g** Relative intensity of the Raman signals of R6G, CuPc and PPP on different substrates, normalized to the signals on SiO_2_/Si. **h** Raman mapping of the intensity of the G band and the characteristic peaks of R6G on P-GQDs. The scale bars are 10 μm in **h**. The unit of the colour bars are a.u. in h

The P-GQDs produced on SiO_2_/Si were directly used as the SERS substrate without post-growth transfer (Fig. 3a). Dipping, dropping and thermal evaporation are commonly used methods to deposit target molecules on SERS substrates^8,17,34^. Here, in order to avoid the uncertainty arisen from different numbers of adsorbed molecules or undesired molecule aggregation, we used vacuum thermal evaporation to deposit 0.2 nm thick R6G, copper phthalocyanine (CuPc), or Protopphyrin IX (PPP) molecules on the testing substrates. After deposition, AFM studies (Supplementary Fig. 12) revealed no molecular aggregation, thus the number of molecules in the beam spot was similar for each Raman test.

The Raman signal was collected by a HORIBA XploRA Raman spectrometer with a 532 nm laser and a 50× objective lens. Figures 3b–d show the Raman spectra of R6G, CuPc and PPP on bare SiO_2_/Si, S-GQDs produced by hydrothermally cutting graphene oxide (Supplementary Note 2)^35^, HOPG, graphene produced by chemical vapor deposition (CVD) and P-GQDs, respectively. Compared with the signal on bare SiO_2_/Si (Fig. 3e–g), no obvious enhancement of the characteristic peaks of R6G, CuPc, PPP is obtained on S-GQDs or HOPG. The enhancement factors relative to the signals on SiO_2_/Si are 59 (1648 cm^−1^ R6G), 20 (1542 cm^−1^ CuPc) and 16 (1490 cm^−1^ PPP) on CVD graphene, comparable to the values reported previously^8^, suggesting a large enhancement effect of graphene. The strongest enhancement occurs on the P-GQDs with enhancement factors up to 437 (1648 cm^−1^ R6G), about two orders higher than that on S-GQDs and about 7 times higher than that on CVD graphene, indicating that the P-GQDs are a more efficient SERS substrate than conventional graphene sheets. Moreover, we measured Raman mapping of the characteristic peaks of R6G (Fig. 3h), CuPc (Supplementary Fig. 13), PPP (Supplementary Fig. 14) on the P-GQDs in a 2500 μm^2^ area. The homogeneous contrast of the characteristic peaks and the G band demonstrate the reliability of the P-GQDs as the SERS substrate and the fact that both the P-GQDs and the deposited molecules are uniform.

### Mechanism of Raman enhancement with P-GQDs

**Fig. 4:**
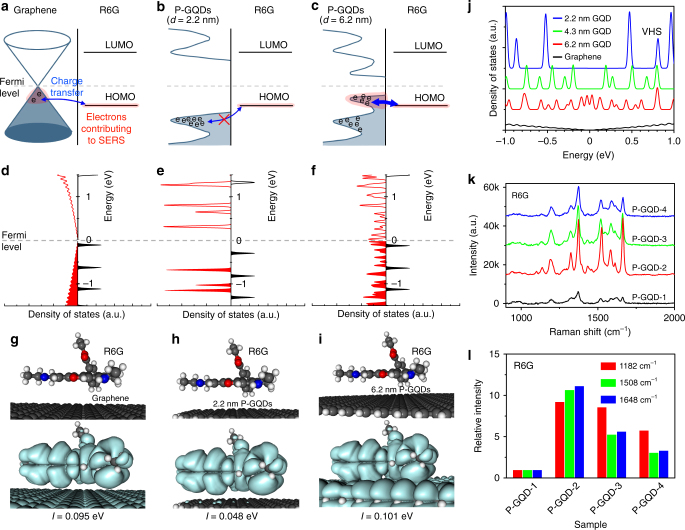
The proposed enhancement mechanism and the size-dependent Raman enhancement for R6G. **a**–**c** The ground state charge transfer between R6G and graphene, GQDs (2.2 nm), or GQDs (6.2 nm). The electron states in the R6G/graphene (or GQD) system, which have the possibility to contribute to the surface-enhanced Raman spectroscopy (SERS), are marked by red shadows. **d**–**f** The calculated density of state (DOS) and **g**–**i** the calculated molecular orbital (at the HOMO level of R6G) densities of R6G/graphene, R6G/GQDs (2.2 nm), and R6G/GQDs (6.2 nm), respectively. (**g**–**i**) The atomic models used in the density functional theory (DFT) calculations and the calculated charge transfer integrals (*I*). **j** The calculated DOS of a graphene and GQDs with different diameters near Fermi level. **k** Raman spectra of thermally evaporated R6G on P-GQDs with different sizes. **l** Relative intensity of the characteristic peaks of R6G on P-GQDs with different sizes, normalized to the signals on SiO_2_/Si. The largest enhancement of R6G signal occurs on P-GQD-2

Until now, two mechanisms of Raman enhancement have been proposed, namely the electromagnetic mechanism (EM) and the chemical mechanism (CM)^36^. Generally, the EM arises from the localized electromagnetic field, which enhances the Raman signals because of the localized surface plasmon resonance effect. The CM is attributed to charge transfer between target molecules and the substrate^37,38^. Considering the smooth surface, high optical transmission of up to 97% and the surface plasmon in the range of terahertz rather than in the visible range^39,40^, the Raman enhancement on graphene is probably attributed to CM rather than EM^41^. Ab initio DFT calculation of R6G on graphene (GQDs) (Fig. 4a–i, Supplementary Figs 15-20) shows that the Fermi level of graphene (GQDs) is different from that of R6G before contact. Charge transfer occurs when contacted until an equilibrium is achieved, leading to re-alignment of the band structure. In the R6G/graphene system (Fig. 4a, d, Supplementary Fig. 15), the highest occupied molecular orbital (HOMO) level of R6G is located near the Fermi level (at −0.08 eV), close to that of graphene^42,43^. Under laser irradiation, Raman scattering is generated by three steps according to the Feynman diagram (Supplementary Fig. 21), which are excitation of the electron in the ground state by the incident light, coupling of the excited electron to the phonon, and radiation of the scattered light when the electron relaxes to the ground state^41^. Enhancing any step of the Feynman process can induce the enhancement of the Raman signals. As a result of the ground-state charge transfer between graphene and R6G (Supplementary Note 3, Supplementary Fig. 22), the electrons near the HOMO in the R6G/graphene system (marked by red shadow in Fig. 4a) have the possibility to contribute to the Raman scattering, thus more electrons are involved in the Raman scattering process of R6G, leading to enhancement of electron−phonon coupling (the second step of the Feynman process)^41^. This, as well as a remarkable suppression of the self-absorption effect of R6G on graphene, results in an obvious Raman enhancement of R6G on graphene^34,41^. Moreover, the charge transfer leads to a higher polarizability. As a result, the displacement of charge density easily occurs under the external light, which also probably leads to the higher Raman scattering cross-section.

Compared with conventional graphene sheets, GQDs have a different band structure as demonstrated by STS (Fig. 2h) and ab initio DFT calculations (Fig. 4j, Supplementary Table 1). We calculated the DOS of the GQDs with different diameters, ranging from 1.14 to 6.17 nm, as well as the perfect graphene as a reference. VHS peaks occur in the DOS of GQDs due to quantum confinement effects. In consideration of the poly-dispersed size, the first VHS of the P-GQD-2 (6–15 nm) broadens to 0.08 eV. Thus, the VHS will not remarkably broaden when averaged over all the GQDs in an ensemble. The band gap, which is defined by the edges of the valence band maximum and the conduction band minimum, increases monotonously with the decreasing of the diameter of the GQDs^30,44^. In the vicinity of the Fermi level, the DOS of the VHS is higher than that of graphene. The significant increase of the DOS near the VHS points leads to strong light-matter interactions and enhances the ground-state charge transfer between GQDs and molecules, involving more electrons in the Raman scattering (Fig. 4c) in the case that the VHS matches the HOMO of molecules^45^. As another possible enhancement origin, the significant increase of the DOS at the VHS also probably enhances excited-state charge transfer (Supplementary Fig. 22). Efficient resonance electron transition from the HOMO level of GQDs to the lowest unoccupied molecular orbital (LUMO) level of molecule occurs if the energy gap between these levels matches the excitation energy of the laser^46^. Moreover, P-GQDs, as a zero-dimensional structure, have more hydrogen-terminated edges^12^, and the hydrogen-terminated bonds may promote efficient charge transfer and enable the enhancement of the Raman signals^11,12,17,47^. These factors discussed above may together lead to the higher Raman enhancement efficiency on P-GQDs than that on conventional graphene sheets as shown in Fig. 3b–d.

The CM is a short-range effect occurring at the molecular scale, and the distance between the substrate and target molecules is of great importance^48^. Functional groups, especially impurities on S-GQDs, significantly reduce the charge transfer and result in a poor enhancement effect of the R6G signal (Supplementary Figs. 23 and 24, Supplementary Note 4). Defects also have a large impact on the enhancement effect. Previous studies showed that oxygen defects produced by UV/ozone on graphene increased the enhancement effect^9^, while C–O, C=O defects on the GQDs produced by electrochemical oxidation decreased the enhancement effect^12^. To clarify the defect effect, we measured the Raman signal of R6G on P-GQDs and exfoliated graphene before and after oxygen plasma treatment. Both plasma treated samples have a slightly weaker enhancement effect (Supplementary Figs. 25-28), indicating that the oxygen defects generated by plasma cannot increase the Raman enhancement. Therefore, the features of P-GQDs such as high crystallization, low defect density, atomically clean surface and accessible edges all appear to be necessary for an efficient SERS substrate.

### Quantum dot size-dependent Raman enhancement

More importantly, the efficiency of charge transfer strongly depends on the energy alignment between the orbitals of GQDs and target molecules. We calculated the energy alignment of R6G on graphene and on GQD (2.2 nm) and GQD (6.2 nm). In the case of R6G/GQD (2.2 nm) (Fig. 4b, e, Supplementary Fig. 17), the HOMO of GQD (−0.68 eV) mismatches with the HOMO of R6G (−0.29 eV), and the calculated molecular orbital (at the HOMO level of R6G) localizes on R6G (Fig. 4h, Supplementary Fig. 18), implying a low efficiency of ground-state charge transfer. In the case of R6G/GQD (6.2 nm) (Fig. 4c, f, Supplementary Fig. 19), increasing the GQD diameter decreases the band gap and the VHS peaks become closer with each other. As a result, the HOMO of GQD (−0.03 eV) appears near the Fermi level, and matches the HOMO of R6G (−0.05 eV). The calculated molecular orbital (at the HOMO level of R6G) spreads over the whole system (Fig. 4i, Supplementary Fig. 20) and has better overlap compared with the R6G/graphene (Fig. 4g). This allows for a highly efficient ground-state charge transfer involving more electrons in the Raman scattering. In the case of R6G/GQD ( > 6.2 nm), further increasing the GQD diameter reduces the DOS at the VHS, leading to weaker light-matter interaction and lower charge transfer efficiency.

To quantitatively assess the charge transfer^49^, we calculated charge transfer integrals (*I*) between GQDs (or graphene) and R6G (Supplementary Table 2, Supplementary Note 5). The R6G/GQD (6.2 nm) system has the largest integral, in good agreement with the above results. Experiments also reveal similar size-dependent enhancement behavior (Fig. 4k, l). P-GQD-2, which has appropriate diameters to meet the requirement of both energy matching and high DOS at VHS, displays the highest enhancement effect for R6G. Therefore, the Raman enhancement effect depends on the GQD diameter, and GQDs with appropriate diameters are required for achieving highly efficient Raman enhancement.

**Fig. 5:**
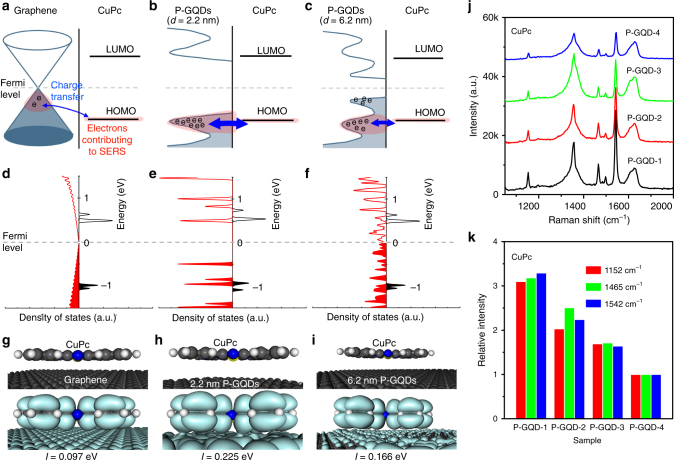
The size-dependent Raman enhancement for CuPc. **a**–**c** The ground state charge transfer between CuPc and graphene, GQDs (2.2 nm) or GQDs (6.2 nm). The electron states in the CuPc/graphene (or GQD) system, which have the possibility to contribute to the SERS, are marked by red shadows. **d**–**f** The calculated DOS and **g**–**i** the calculated molecular orbital (at the HOMO level of CuPc) of CuPc/graphene, CuPc/GQDs (2.2 nm), and CuPc/GQDs (6.2 nm), respectively. (**g**–**i**) The atomic models used in the density functional theory (DFT) calculations and the calculated charge transfer integrals (*I*). **j** Raman spectra of thermally evaporated CuPc on P-GQDs with different sizes. **k** Relative intensity of the characteristic peaks of CuPc on P-GQDs with different sizes, normalized to the signals on P-GQD-4. The largest enhancement of CuPc signal occurs on P-GQD-1

In addition to R6G, we also calculated energy alignments of CuPc (Fig. 5a–f, Supplementary Figs 29–31, Supplementary Note 6) on graphene, GQD (2.2 nm) and GQD (6.2 nm), respectively. CuPc has a different set of energy levels. The calculated HOMO level of CuPc matches well with all of these substrates. In consideration of the small size of the 2.2 nm GQD, the DOS at the VHS is not only significantly higher than the DOS of graphene, but also higher than that of GQDs with larger diameters, leading to better overlap of the molecular orbital (at the HOMO level of CuPc; Fig. 5g–i), higher charge transfer integral (Supplementary Table 2), more electrons involved in the Raman scattering and a larger enhancement effect. In agreement with the calculation, experiments exhibit the largest enhancement of CuPc signal on P-GQD-1 (Fig. 5j, k), different from the R6G results. Therefore, the appropriate diameter for achieving strong Raman enhancement depends on the target molecules, owing to the different energy alignment of different molecules.

### Application of P-GQDs in SERS substrates

**Fig. 6:**
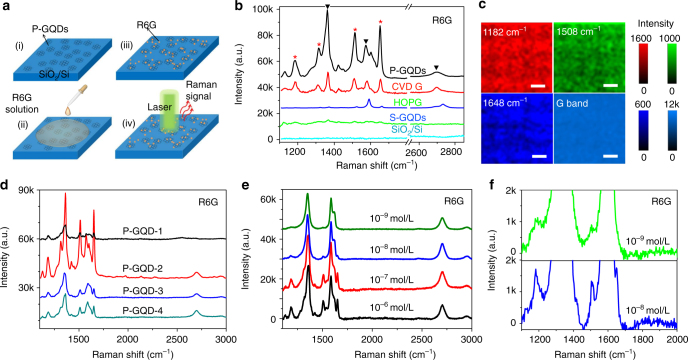
Application of P-GQDs for detecting R6G in ethanol. **a** Schematic of the experimental procedure. **b** Raman spectra of R6G (10^−5^ mol L^−1^) on SiO_2_/Si, S-GQDs, HOPG, transferred CVD graphene and P-GQD substrates. Black arrows indicate the peaks from P-GQDs, and red stars indicate the peaks from R6G. **c**, Raman mapping of the intensity of the G band and the characteristic peaks of R6G (10^−5^ mol L^−1^) on P-GQDs. **d** Raman spectra of R6G (10^−5^ mol L^−1^) on P-GQDs with different sizes. **e** Raman spectra of R6G with concentrations from 1 × 10^−5^ to 1 × 10^−9^ mol L^−1^ on P-GQDs. **f**, Magnified Raman spectra of R6G with concentrations of 1 × 10^−8^ mol L^−1^ and 1 × 10^−9^ mol L^−1^ on P-GQDs. The scale bars are 10 μm in **c**. The unit of the colour bars are a.u. in c

Finally, the P-GQDs were applied as the SERS substrates for detecting R6G molecules in ethanol (Fig. 6a). A 10 µL ethanol solution containing 10^−5^ mol L^−1^ R6G was dropped on the P-GQDs and dried. Figure 6b shows Raman spectra of R6G on bare SiO_2_/Si, S-GQDs produced by hydrothermally cutting graphene oxide, HOPG, CVD graphene and P-GQDs. The characteristic peaks have no obvious shift, indicating R6G molecules are adsorbed as individual molecules rather than dimers^50^. No obvious peaks of R6G appear on bare SiO_2_/Si; while small peaks of R6G are detected on S-GQDs and HOPG with normalized enhancement (relative to the signal on SiO_2_/Si) lower than 200. On CVD graphene, the enhancement factor increases to 1281 (1182 cm^−1^). The strongest enhancement occurs on the P-GQDs with enhancement factors up to 2370 (1182 cm^−1^) (Supplementary Fig. 32). Compared with thermally evaporated R6G, the higher enhancement factor should be attributed to more R6G molecules adsorbed from solution as a result of the large amount of accessible edges of P-GQDs. Moreover, owing to the quasi-equilibrium growth process of P-GQDs, some dangling bonds exist on the edges, which probably act as hot spots to effectively adsorb or trap more molecules^17,51^. To clarify the reliability of the P-GQDs in the applications, we measured Raman mapping of the characteristic peaks of R6G on P-GQDs. The relatively homogeneous intensity over a large area (Fig. 6c) indicates the high reliability of the P-GQDs in practical applications. Finally, to improve the enhancement performance, P-GQDs were prepared at different temperatures by qe-PECVD. By analyzing the characteristic R6G peaks at the 1182, 1508, and 1648 cm^−1^, we found that P-GQD-2 grown at 615 °C displays the highest enhancement effect for R6G (Fig. 6d), consistent with the thermal deposition results.

Owing to the strong intrinsic enhancement as well as the efficient adsorption of the target molecules, P-GQDs can be applied in detecting a low concentration analyte. R6G solutions with different concentrations were dropped on P-GQD-2. In the Raman spectra (Fig. 6e), characteristic peaks of R6G are clearly observed when the concentration is 1 × 10^−7^ or 1 × 10^−8^ mol L^−1^, and some signals from R6G can still be detected even when the concentration decreases to 1 × 10^−9^ mol L^−1^ (Fig. 6f). Supplementary Fig. 33 shows the Raman intensity of the characteristic peaks under different concentrations. The intensity increased with the concentration when the concentration is low, while in the case of high concentration, a much smaller increase of the intensity is observed, owing to the first-layer effect^52^. Therefore, the enhancement from more adsorbed molecules is dependent on the concentration. At high concentrations or with large numbers of target molecules, more adsorbed molecules could not lead to much additional enhancement; while at low concentrations or with small numbers of target molecules, the enhancement increases. As a result, at ultra-low concentration (1 × 10^−9^ mol L^−1^), the intrinsic enhancement of P-GQDs along with additional molecules adsorbed leads to the enhancement about one order-of-magnitude larger than than the result on conventional mechanical exfoliated graphene^8^, indicating the high sensitivity of P-GQDs as a SERS substrate.

## Discussion

In this article, we develop a qe-PECVD method to produce high-quality ultra-clean GQDs directly on SiO_2_/Si with size down to 2 nm without catalysts. STM and HRTEM measurements show that the P-GQDs have high crystallinity, atomically clean surfaces without functional groups and impurities, which normally exist in S-GQDs. STS result reveals the semiconducting nature of P-GQDs and VHS in the electronic DOS, in good agreement with ab initio DFT calculations. These properties probably induce strong light-matter interaction and enhanced charge transfer between P-GQDs and target molecules. This, as well as the high quality, ultra-clean surface, and large amount of accessible edges as absorption sites make P-GQDs an efficient SERS substrate with enhancement factors up to 2370, about 1.85 folds larger than that on CVD graphene. The Raman signal of R6G is detectable even at concentrations as low as 10^−9^ mol L^−1^, about one order-of-magnitude lower than previous results on mechanically exfoliated graphene, indicating its great potential for application as a new type of sensitive carbon-based SERS substrates. More importantly, both experiment and DFT calculation reveal a size-dependent enhancement behavior, and the largest enhancement occurs on P-GQD-2 for R6G, and on P-GQD-1 for CuPc, indicating that the appropriate diameter depends on the type of the target molecule. This finding extends fundamental understanding of the Raman enhancement by GQDs, and would be valuable for developing highly sensitive GQD-based SERS substrate. Due to the strong light-matter interaction, this high-quality ultra-clean GQD is also a potential candidate material for future graphene-based photonics and optoelectronics.

## Methods

### Growth of P-GQDs

P-GQDs were prepared by qe-PECVD. A SiO_2_/Si substrate was washed by ultrasonication in acetone, ethanol and water, respectively, and was placed in a 2-inch quartz tube at the center of the furnace (MTI Corporation, OTF-1200X). The furnace was heated to the growth temperature under a constant pure methane gas flow of 5 sccm (120 mTorr), and then methane plasma was generated upstream by a remote radiofrequency plasma generator (K-Mate, VERG-500, 20 W). After 10 min growth, the furnace was cooled to room temperature under ambient H_2_. The P-GQDs were obtained on the SiO_2_/Si.

### Characterization

The samples were measured by AFM (Multimode 8, Bruker, tapping mode), HRTEM (Tecnai G2 F20 S-Twin, acceleration voltage: 200 kV), EDS (equipped on HRTEM) and XPS (Perkin-Elmer PHI 5300 with 250 W Mg Kα source, 1253.6 eV). For HRTEM measurement, the P-GQDs were transferred to the copper grids by using poly-methyl-methacrylate (Supplementary Note 7). The STM measurements were carried out in a custom-built multi-chamber ultra-high vacuum system housing an Omicron LT-STM in the analysis chamber with a base pressure better than 1.0 × 10^−10^ mbar. All the STM images were recorded in constant current mode at liquid nitrogen temperature (77 K) using electrochemically etched tungsten tips. The STS data were acquired using a lock-in amplifier by applying a small sinusoidal modulation to the tip bias voltage (typically 30 mV at 600 Hz). All STM images were processed using WSxM.

### Raman measurement

A 0.2 nm thick layer of R6G (Sigma-Aldrich), CuPc (Sigma-Aldrich) and PPP (Sigma-Aldrich) molecules were deposited on the surface of the test substrates by thermal evaporator (Nexdep, Angstrom Engineering) at 10^−5^ Torr. In practical applications, 10 µL of 10^−5^ mol L^−1^ R6G in ethanol was dropped on the test substrates, and dried in air. Raman spectra were collected by a HORIBA XploRA Raman spectrometer with a 532 nm wavelength excitation laser and an optical grating (1200 lines per mm). The laser beam was focused by a 50× objective lens, resulting in a spot size of around 2 μm in diameter. The acquisition time was 10 s for each spectrum.

### Simulation Method

The ab initio DFT calculation was conducted by RESCU (Real space Electronic Structure CalcUlator), which is a powerful Kohn-Sham density functional theory solver^53^. The generalized gradient approximation was used for the exchange-correlation potential by Perdew, Burke and Ernzerhof^54^. A linear combination of atomic orbital (LCAO) method is used to expand physical quantities and the standard norm-conserving pseudopotentials are used to define the atomic core states. The double zeta double polarization functions (DZDP) basis set is used for each atom in our calculation. A self-consistent field procedure was carried out until the global charge variation was less than 10^−4^. The distance between the molecule and the graphene (or GQD) was optimized by generalized gradient approximation (GGA). The wave function and electron density were expanded by double numeric quality basis set with DZDP. The tolerances of energy and charge were 1 × 10^−4^ Ha and 1 × 10^−4^ electrons. GQDs with both zigzag and armchair shaped edge structures have been investigated. We calculated R6G (63 atoms), CuPc (57 atoms) on two GQDs (ZZ09: 180 atoms, 22.2 Å diameter; ZZ25: 1092 atoms, 61.7 Å diameter). As a comparison, we also calculated R6G and CuPC on a graphene plane (800 atoms, 49.4 × 42.7 Å^2^, a periodical boundary condition). The R6G molecule was adsorbed onto the GQD with the optimized geometrical condition based on previous works^55^, especially the dihedral angle between the phenyl group and xanthene rings in R6G was set to 72^o^ ^34^. The optimized values for the distance between the absorbed R6G and corresponding substrates were 4.053 Å (ZZ09), 3.880 Å (ZZ25), and 5.133 Å (Graphene). The optimized geometrical condition with the CuPc being absorbed at the hollow site on GQD and graphene was adopted^56^. The optimized values for the distance between the absorbed CuPc and the GQD/graphene substrates were 3.588 Å (ZZ09), 3.741 Å (ZZ25), and 4.341 Å (Graphene).

### Data availability

The data that support the findings of this study are available from the corresponding author upon request.

## Electronic supplementary material

Supplementary Information
