# Supplementary material for: Raman enhancement on ultra-clean graphene quantum dots produced by quasi-equilibrium plasma-enhanced chemical vapor deposition
Source: Nat Commun. 2018 Jan 15;9:193. doi: 10.1038/s41467-017-02627-5 (PMC5768689; doi:10.1038/s41467-017-02627-5)
Supplement: Supplementary file 1 — Supplementary Information [file 41467_2017_2627_MOESM1_ESM.pdf]

Supplementary Note 1. Detailed Description of the qe-PECVD

Previous research has demonstrated that there is a competition between the effects of graphene etching and growth in PECVD, and high efficient catalyst-free growth of graphene crystals (Supplementary Fig. 2a) only takes place in an equilibrium state at critical temperature (T_c)[1,2]. The competition can be expressed by the following equation:

$$CH_4(g) \underset{\text{etching}}{\overset{\text{growth}}{\rightleftharpoons}} \text{Graphene(s)} + 2H_2(g) - Q \qquad (1)$$

In PECVD, the plasma decomposes precursor molecules into highly reactive C_xH_y species (radicals, ions, atoms) as the feedstock for graphene growth[3,4]. Reactive C_xH_y species result in nucleation of graphitic clusters at a temperature higher than T_c, and introduce structural defects on the edges, which prevent the growth of graphene crystals. Thus, disordered carbon films are grown by PECVD at a temperature higher than T_c (Supplementary Fig. 3). On the other hand, atomic hydrogen is commonly accepted as an etchant during carbon growth[5]. Previous research has demonstrated that H_2 plasma can etch graphene from the edges[5]. H species also exist in CH_4 plasma. At a temperature lower than T_c, the etching effect of H species dominates the PECVD process (Supplementary Fig. 4), thus no graphene (Figure 1b) can be obtained.

At T_c, a critical equilibrium state between the effects of graphene etching and growth is achieved. The moderate etching effect of H species suppresses the nucleation of graphitic clusters and removes edge defects, keeping the edges active and atomically smooth (Supplementary Fig. 2b) during the whole growth process. As a result, the crystal edge growth mode dominates (the nucleation is suppressed), and high quality hexagonal graphene crystals are produced directly on SiO_2/Si.

In this study, P-GQDs are produced at a temperature slightly higher than T_c. In a quasi-equilibrium state between the effects of graphene etching and growth, both nucleation of GQDs and crystal growth can be realized, resulting in large amount of high quality GQDs grown on SiO_2/Si.

Supplementary Note 2. The method to prepare S-GQDs

Two types of S-GQDs were used.

One type of the S-GQDs was produced via a bottom-up approach by polymerization and carbonization reactions[6,7]. Citric acid monohydrate (1.26 g) and ethylenediamine (1.608 mL) was dissolved in DI-water (30 mL) and the mixture was stirred vigorously for 10 min. Then obtained transparent solution was transferred to a Teflon-lined stainless-steel autoclave (50 mL capacity) and sealed to heat at 200°C. After reacting for 5 h, the autoclave was cooled to room temperature by water and the brown-black products were obtained. The average size was about 5 nm. The sample was dissolved in water, but DMF and other molecules may exist in the S-GQDs.

The other type of S-GQDs was produced via a top-down approach by cutting the graphene oxide sheets using hydrothermal reactions[8]. Graphene oxide sheets were prepared from natural graphite powder by a modified Hummers method. Graphene sheets were obtained by thermal deoxidization of GO sheets in a tube furnace at 200–300 °C for 2 h in a nitrogen atmosphere. Graphene sheets (0.05 g) were oxidized in concentrated H_2SO_4 (10 mL) and HNO_3 (30 mL) for 15–20 h under ultrasonication. The mixture was then diluted with deionized water and filtered through a 0.22-μm microporous membrane to remove the acids. Purified oxidized graphene sheets (0.2 g) were re-dispersed in deionized water and the pH was tuned to 8 with NaOH. The suspension was transferred to a poly(tetrafluoroethylene) (Teflon)-lined autoclave and heated at 200 °C for 10 h. After cooling to room temperature, the resulting black suspension was filtered through a 0.22-μm microporous membrane and a brown filter solution was separated. The colloidal solution was further dialyzed in a dialysis bag overnight and then S-GQDs were obtained. The average size was about 5-13 nm, and the sample was dissolved in water.

Supplementary Note 3. Charge transport in the Raman scattering of R6G on graphene

Supplementary Fig. 22a shows the Raman scattering process of a R6G molecule, which includes three steps. In the first step, the electrons in the HOMO of R6G are excited to the LUMO of R6G by 532 nm laser, forming electron-hole pairs. In the second step, the excited electrons couple to the phonons and relax to vibrational states.

In the third step, the electrons relax to the HOMO of R6G and the electron-hole pairs recombine.

On graphene, the charge transfer between target molecules and the substrate largely enhances the Raman scattering of the molecules. There are two widely accepted charge-transfer models, which are the ground-state charge transfer[9] and the excited-state charge transfer[10]. In the case of the ground-state charge transfer model, the resonance is not necessary for the Raman enhancement. Charge transfer occurs when the substrate and the molecule are in the ground state which does not need the aid of the light radiation. In the case of the excited-state charge transfer model, the maximum enhancement is usually associated with the transition energy to the charge transfer resonance state. The excited-state charge transfer only takes place when the energy gap between graphene (HOMO) and molecules (LUMO) matches the photon energy of the laser[11]. There are no convictive evidences to support this model, while the ground-state charge transfer model can better fit the experimental and simulation results. Thus, the ground-state charge transfer should be the main mechanism, as shown in Fig. 4a.

The electron process in graphene-enhanced Raman scattering is a very complicated issue. When R6G molecules contact with graphene, charge transfer occurs as a result of the different work functions of the graphene and the R6G. With the re-distribution of the electrons between the R6g and the graphene, new equilibrium can be achieved, which gives rise to re-alignment of the band structure. Owing to the strong charge transfer, the molecule cannot be regarded as isolated molecule[12,13]. Instead, the molecule should be a part of the molecule/graphene system. The SERS phenomenon should be related to the whole R6G/graphene system instead of the isolated R6G molecule. In the R6G/graphene system, although the actual electron process of Raman scattering is still not well understood, it is clear that the charge (electrons and holes) at the same energetic level can freely transfer between the R6G and graphene, which actually provides more density of state at the HOMO of R6G, compared with the isolated R6G molecule. The electrons near the HOMO level of the R6G in the system have the possibility to contribute to the SERS, thus more electrons are involved in the Raman scattering process of the R6G, leading to enhancement of the Raman signal.

Supplementary Fig. 22b and 22c provide some of the possible ground-state charge-transfer processes inside the R6G/graphene system, which contribute the SERS. In these

processes, (step 1) the electrons in the HOMO of R6G are excited by the laser, which generates holes in the HOMO and allows (step 2) the ground-state electron transfer from graphene to the HOMO of the R6G. And then, the electrons in graphene are involved in the Raman scattering process of the R6G (step 3~5). Finally, the electrons transfer from the HOMO or vibrational states of the R6G to graphene (step 5 or step 6). Besides ground-state charge transfer, other types of charge transfer probably exist. For instance, the excited-state charge transfer (Supplementary Fig. 22d-f) probably occurs when the energy gap between the HOMO of the graphene and the LUMO of the R6G matches the photon energy (2.33 eV) of the laser[10].

Supplementary Note 4. Raman enhancement of R6G on S-GQDs

To measure the Raman spectra, we dropped the S-GQDs on a SiO_2/Si substrate and dried in vacuum. 10 μL of 10^{-5} mol L^{-1} R6G ethanol solution was dropped on the S-GQDs and dried in air. Raman spectra were characterized by a HORIBA XploRA Raman spectrometer with a 532 nm wavelength excitation laser, and an optical grating (1200 lines/mm). The laser beam was focused by a 50× objective lens, resulting in a spot size of around 2 μm in diameter. The acquisition time was 10 s for each spectrum.

Supplementary Figs 21 and 22 show the Raman spectra of S-GQDs and R6G on S-GQDs. Two types of S-GQDs were used. S-GQDs produced via a bottom-up approach by polymerization and carbonization reactions have poor crystallization, a large amount of impurities, and high content of functional groups like -OH, C=O, C-N, NH[6,7]. Due to the poor quality, characteristic peaks of both GQDs and R6G cannot be observed (Supplementary Fig. 21). S-GQDs produced via a top-down approach by cutting the graphene oxide sheets using hydrothermal reactions[8] have higher quality with better crystallization and functional groups like -OH, C=O, COOH, etc. The S-GQDs are dispersed in water solution, thus it is cleaner than the other type. As a result, Raman signal of R6G can be detected although these peaks are weak (Supplementary Fig. 22), indicating the importance of the quality and cleanliness of the sample in SERS applications.

Supplementary Note 5. Calculation of the charge transfer integral.

The mechanism of the SERS, in our work, is attributed to the enhanced charge transfer between the GQD and the absorbed molecules. The transfer coupling is quantitatively assessed through the calculation of the charge transfer integral for the R6G and CuPC absorbed on the GQD/Graphene substrates, following the strategy developed by Deng *et al.* in their calculation of the coupling matrix element for organic semiconductors[14], which was lately adopted in study on the transport properties of organic semiconductors on two-dimensional materials[15]. In our case, the charge transfer integral (I) for a molecular-on-GQD system is related to the energetic splitting of that level in the absorption system as compared to the isolated neutral molecule and the GQD. Thus the I is given by,

$$I = \frac{1}{2}\sqrt{(E_1 - E_2)^2 - (\varepsilon_1 - \varepsilon_2)^2} \quad (2)$$

where E_1 and E_2 are the two respective highest occupied orbitals for the GQD template and the absorbed R6g or CuPC molecule. And ε_1 and ε_2 are the corresponding highest occupied orbitals for the isolated molecule and GQD template. The quantities used in the Eq.2 were obtained from the band structure calculations with RESCU as indicated above. Supplementary Table 2 shows the obtained charge transfer integrals obtained based on the Supplementary Equation 2.

Supplementary Note 6. Raman enhancement mechanism of the CuPc/GQDs

The electronic structures of CuPc molecule adsorbed on 2 nm GQD, 6 nm GQD and graphene has also been investigated with RESCU. We adopt the general modelling strategy as developed by the previous works[16,17], in which the molecular plane is parallel to the graphene plane and the top site is chosen as the adsorption site for the CuPc molecule on both GQDs and graphene surface. The spin polarization is considered in this calculation[18]. Since the copper is not generally associated with magnetic properties in the case of CuPc, unrestricted spin configuration has been applied[16,17,19]. The density of states for different configurations is shown in Figure 5d-f. The calculation results show that the HOMO of CuPc is mainly dominated by Cu 3d orbital with contributions from C 2p orbital, while the LUMO of CuPc is originated from Cu 3d, C 2p and N 2p orbitals, consistent with the results from the previous works[16-18].

The calculation result shows the Fermi level of graphene (or GQD) is different from that of CuPc before contact. Once the CuPc is adsorbed onto a GQD, charge transfer takes place until an equilibrium is achieved, leading to re-alignment of the band structure. After re-alignment, calculated HOMO level of CuPc matches all of the substrates. 2.2 nm GQD has small dot size. As a result, the DOS at the VHS is higher than that of graphene or GQDs with large diameter. The remarkable increase of the DOS at the VHS leads to strong light-matter interaction and high charge transfer efficiency between 2.2 nm GQD and CuPc molecule. Thus, the enhancement effect on 2.2 nm GQD is stronger than that on graphene and 6.2 nm GQD. In the experiments (Fig. 5h,i), we also find that the strongest enhancement occurs on P-GQD-1 for CuPc, consistent with the calculation. For R6G, the strongest enhancement occurs on P-GQD-2. Therefore, the appropriate diameter for achieving strong Raman enhancement depends on the type of the target molecules, owing to the different energy alignment between molecules and GQDs.

Supplementary Note 7. **Experimental details of preparing the TEM samples.**

5% poly-methyl-methacrylate (PMMA) was spin-coated on the as-grown GQDs/SiO_2/Si substrates at 3000 rpm for 60 seconds and further strengthened by heating at 180°C for a few minutes. The GQDs with PMMA were immersed in 20% hydrofluoric acid until the PMMA/GQDs exfoliated from the substrate. PMMA/GQDs were rinsed several times with DI-water to remove the residual acid. The PMMA/GQDs were then transferred onto a TEM copper grid, and dried at 100°C for a few minutes. The PMMA was removed by acetone vapour, and then the sample was annealed at 400 ºC in 200 sccm Ar for 1 hour.

Supplementary Table 1

Models	Diameter (nm)	Number of atoms for all，C，H	Band Gap(eV)
AC3	1.14	60，42，18	2.46
AC5	2.00	144，114，30	1.53
AC7	2.85	264，222，42	0.61
ZZ05	1.24	72，54，18	1.90
ZZ07	1.73	120，96，24	1.35
ZZ09	2.23	180，150，30	0.99
ZZ11	2.72	252，216，36	0.77
ZZ15	3.70	432，384，48	0.39
ZZ25	6.17	1092，1014，78	0.06

Supplementary Table 1. The diameters, number of atoms, and the calculated band gap for the investigated graphene quantm dot models.

Supplementary Table 2

	ZZ09-R6G	ZZ25-R6G	Graphene-R6G	ZZ09-CuPc	ZZ25-CuPc	Graphene-CuPc
I (eV)	0.048	0.101	0.095	0.225	0.166	0.097

Supplementary Table 2. The calculated charge transfer integrals (*I*) between molecule (R6G or CuPc) and GQD/graphene.

Supplementary Fig. 1

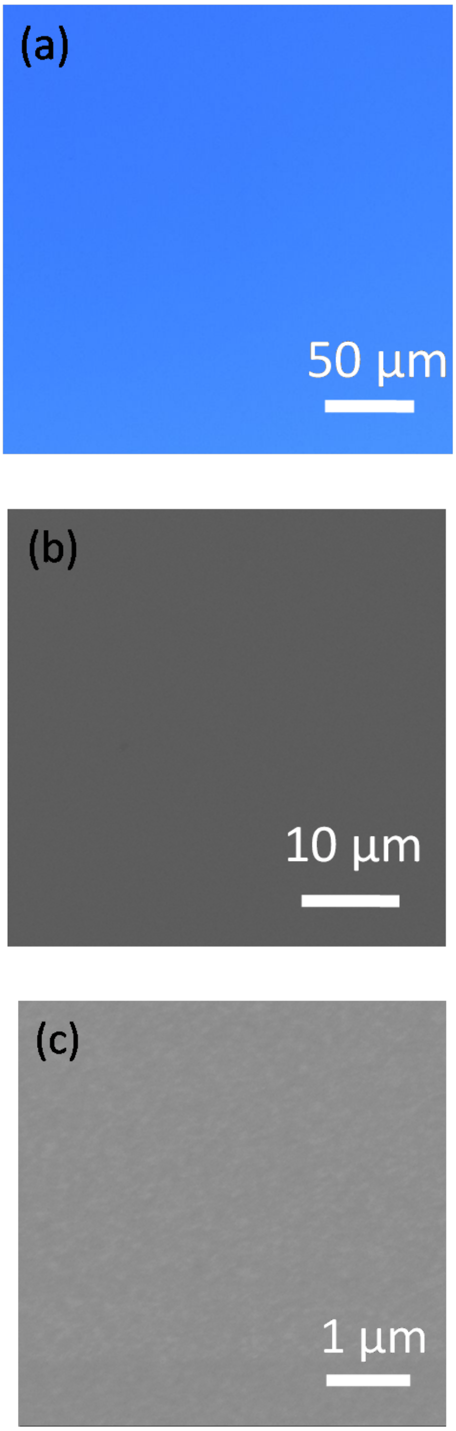

Supplementary Fig. 1. (a) Optical image of the P-GQDs grown on a SiO_2/Si substrate. (b, c) SEM images of the P-GQDs grown on a SiO_2/Si substrate.

Supplementary Fig. 2

(a)

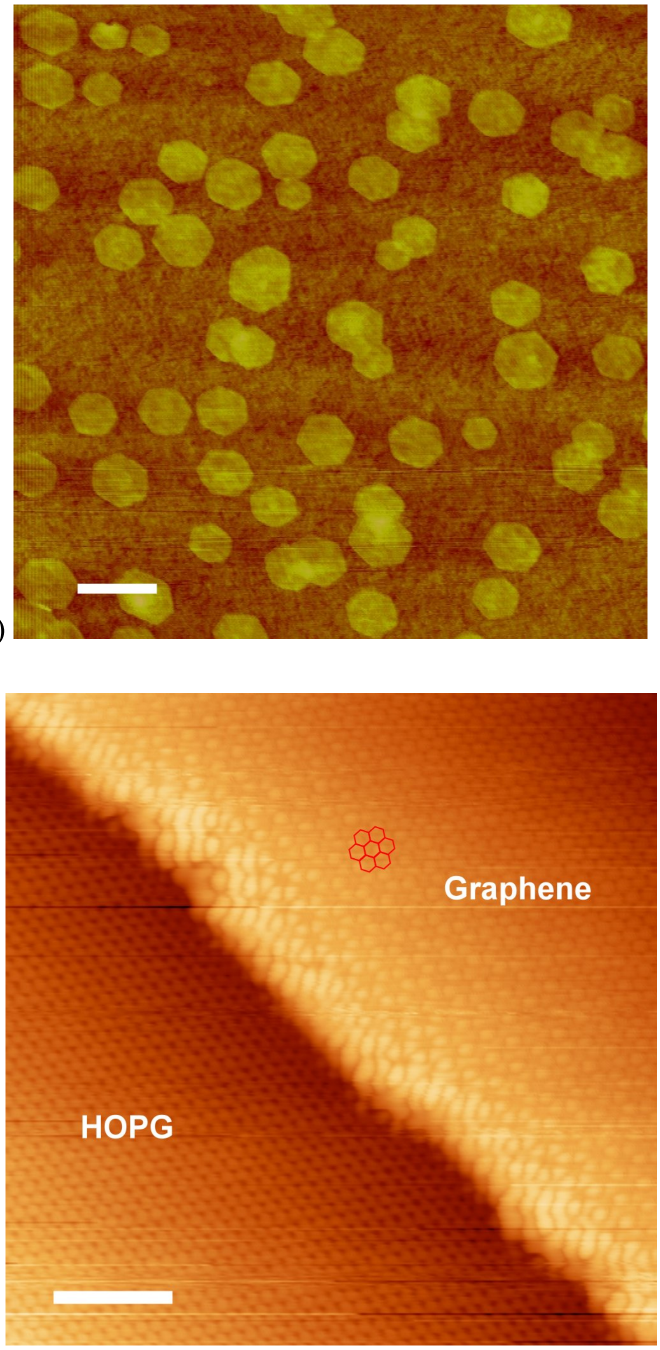

(b)

Supplementary Fig. 2. (a) AFM image and (b) STM image of hexagonal graphene crystals grown in a critical equilibrium state at critical temperature. The graphene lattices are marked by red hexagons. The scale bars are 500 nm in (a) and 2 nm in (b).

Supplementary Fig. 3

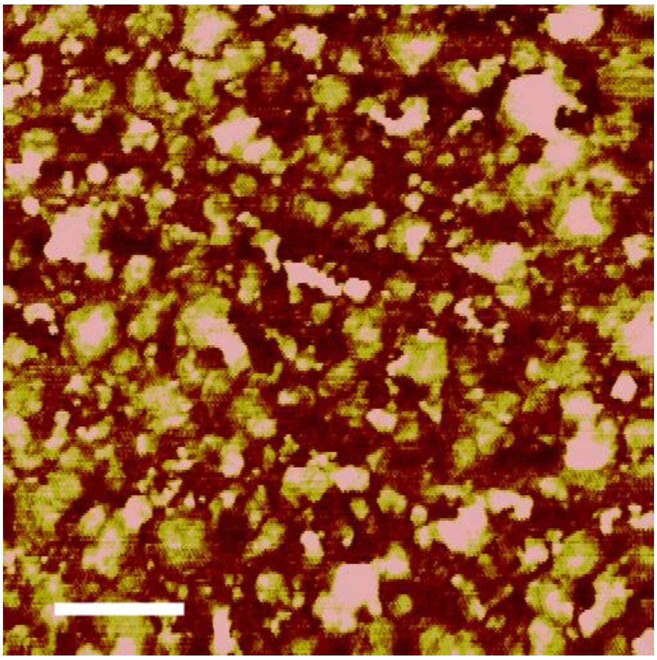

Supplementary Fig. 3. AFM image of a carbon film produced by PECVD on SiO_2/Si at 700 ºC, which indicates that highly disordered carbon film is obtained in a non-equilibrium state at a temperature larger than the critical temperature. The scale bar is 1 μm.

Supplementary Fig. 4

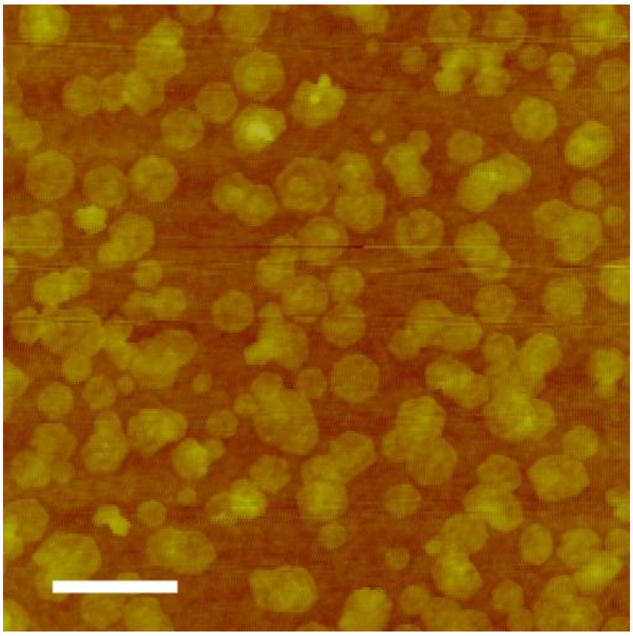

(a)

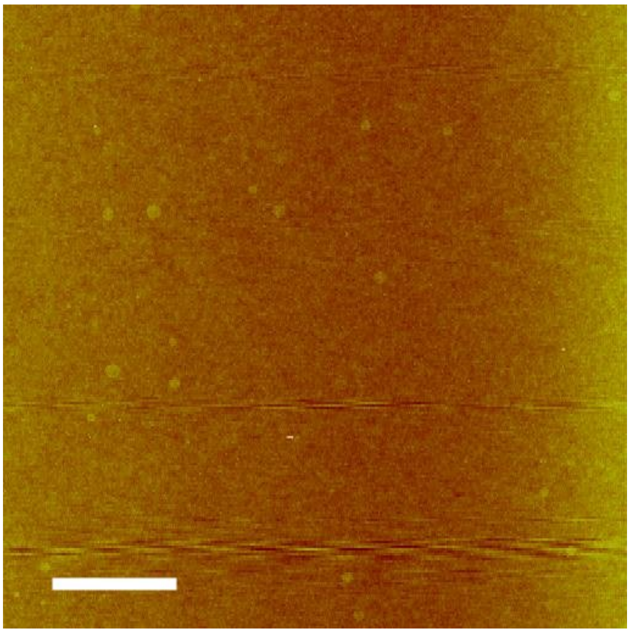

(b)

Supplementary Fig. 4. AFM images of graphene crystals on SiO_2/Si (a) before and (b) after a treatment by CH_4 PECVD at 500 °C. The result shows that the graphene crystals are etched at a temperature under the critical temperature. The scale bars are 1 μm.

Supplementary Fig. 5

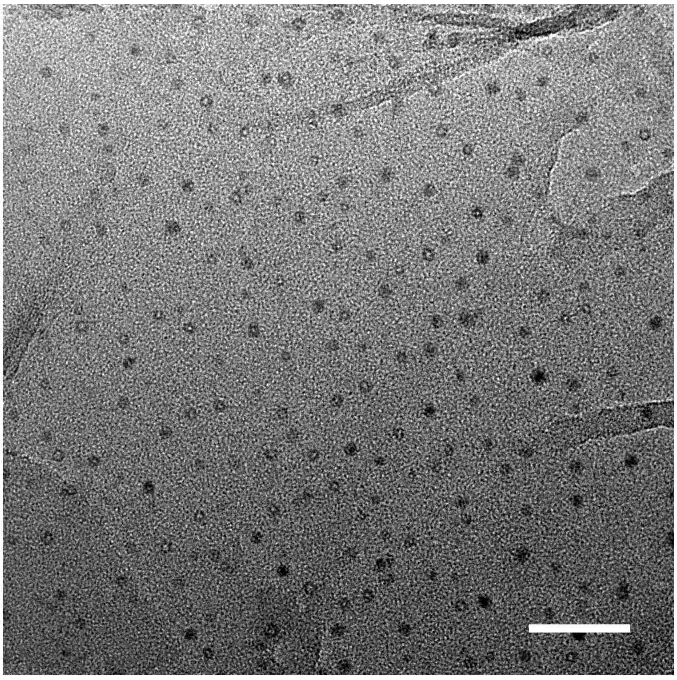

(a)

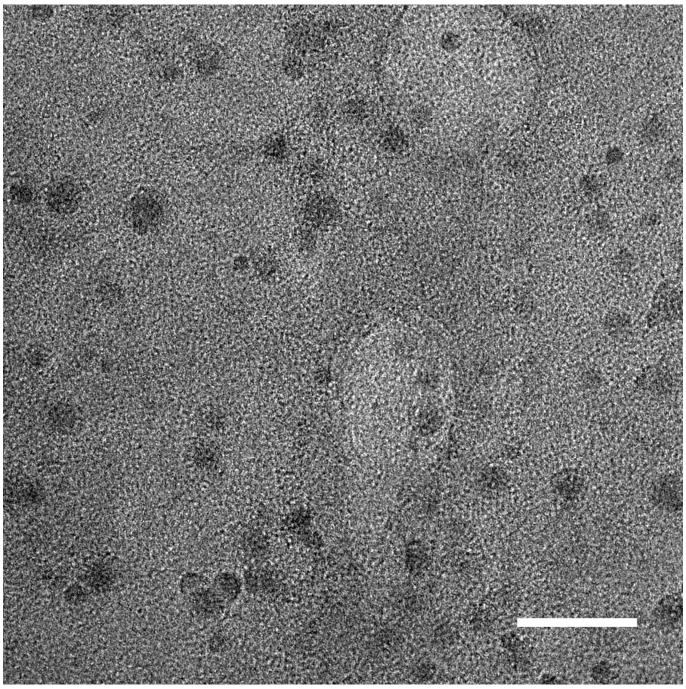

(b)

Supplementary Fig. 5. TEM images of the P-GQD-1 after transferred from the SiO_2/Si substrate to the carbon membrane of a copper grid. The scale bars are 50 nm (a) and 20 nm (b).

Supplementary Fig. 6

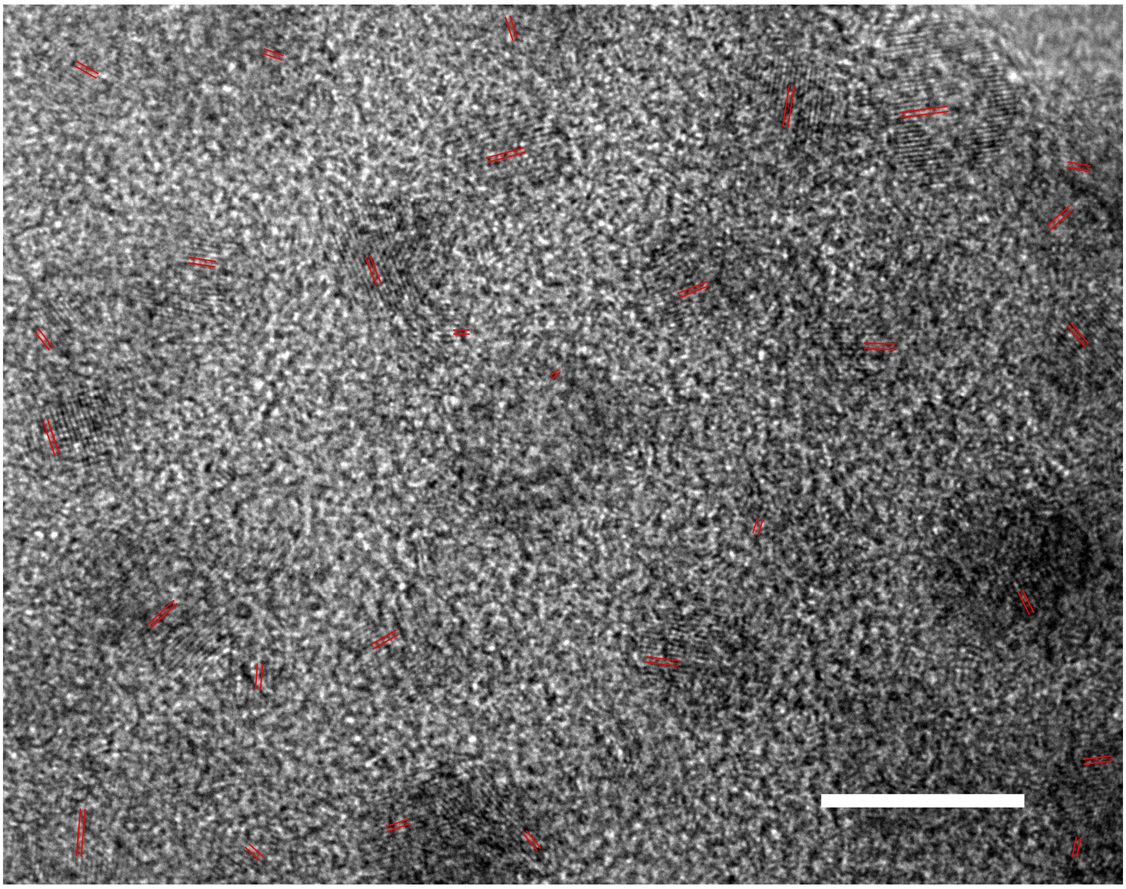

Supplementary Fig. 6. A HRTEM image of the P-GQD-1 after transferred from the SiO_2/Si substrate to the carbon membrane of a copper grid. The lattice fringes are highlighted by red lines. The scale bar is 10 nm.

Supplementary Fig. 7

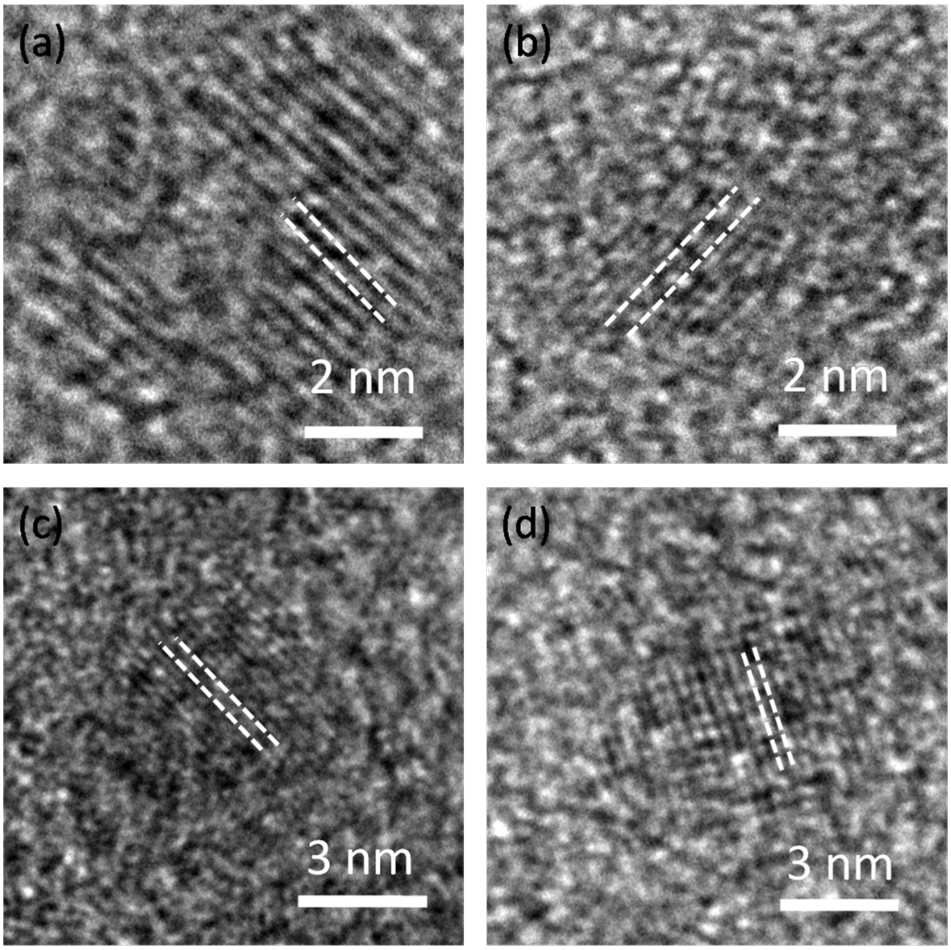

Supplementary Fig. 7. HRTEM images of the P-GQD-1 sample after transferred from the SiO_2/Si substrate to the carbon membrane of a copper grid. (a-d) In the HRTEM images, the lattice fringes are highlighted by white lines.

Supplementary Fig. 8

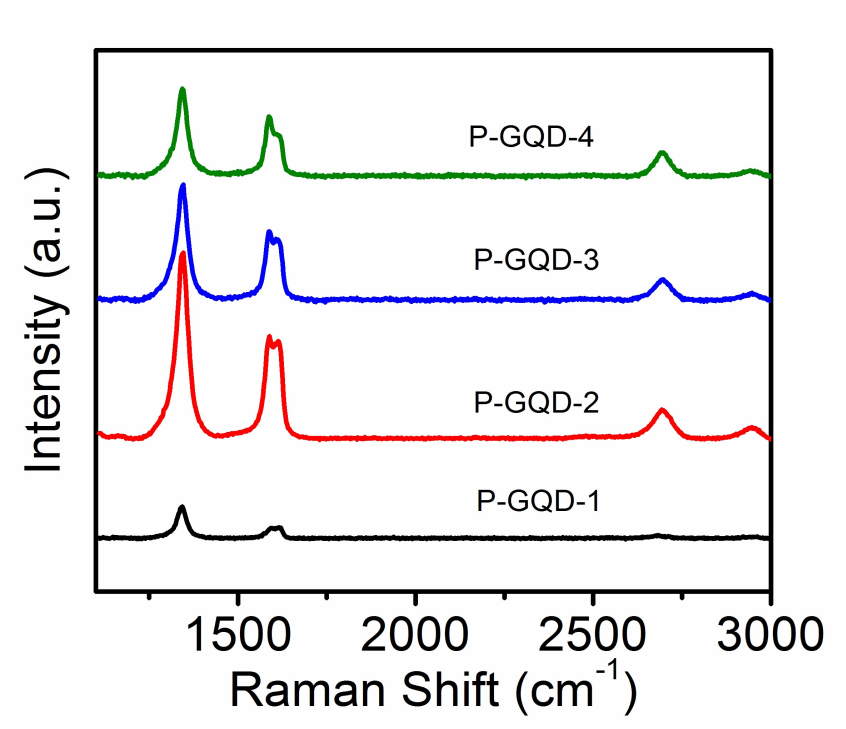

Supplementary Fig. 8. Raman spectra of P-GQDs with different sizes.

Supplementary Fig. 9

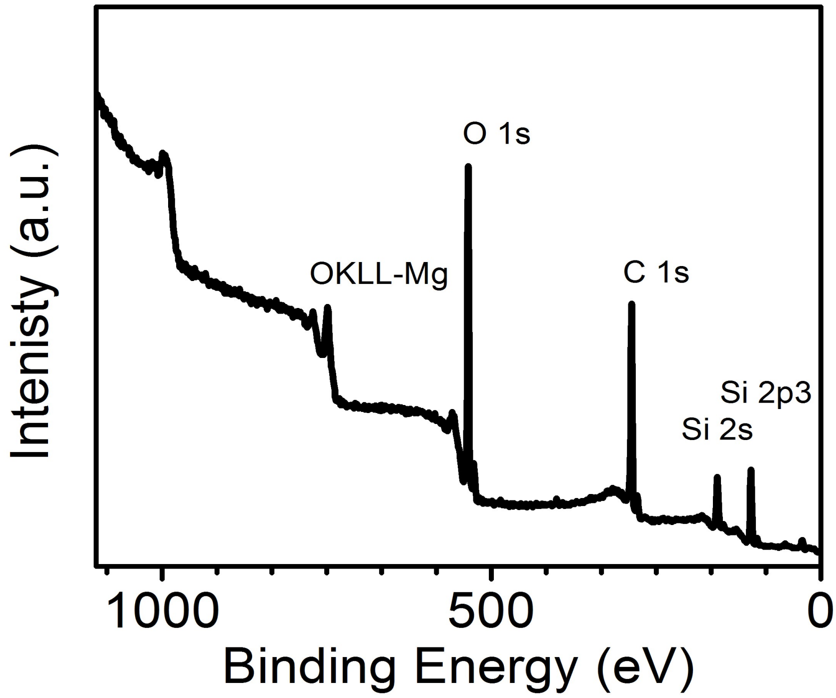

Supplementary Fig. 9. Wide range XPS spectrum of the P-GQDs grown on SiO_2/Si.

Supplementary Fig. 10

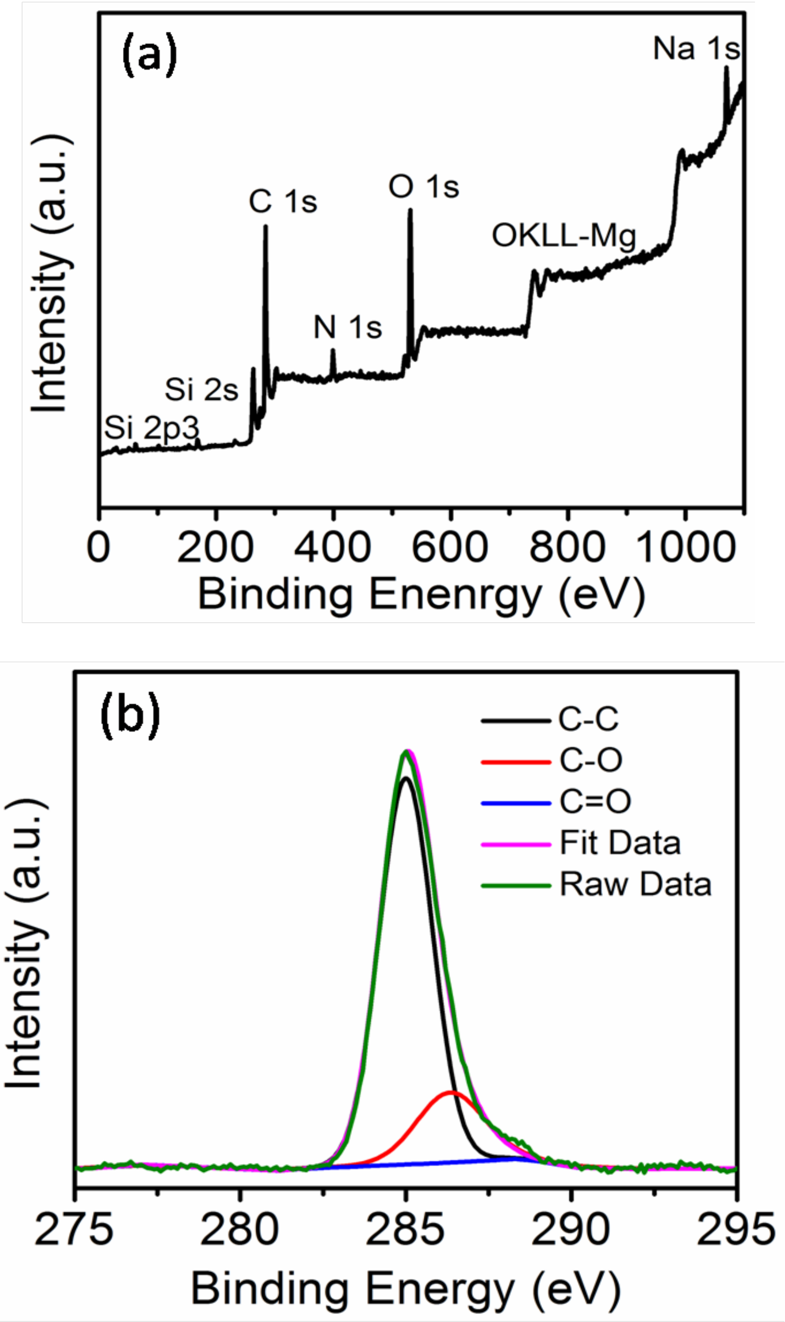

Supplementary Fig. 10. (a) Wide range XPS spectrum of the S-GQDs (produced by cutting the graphene oxide sheets using hydrothermal reactions) on SiO_2/Si. (b) XPS C1s spectrum of the S-GQDs.

Supplementary Fig. 11

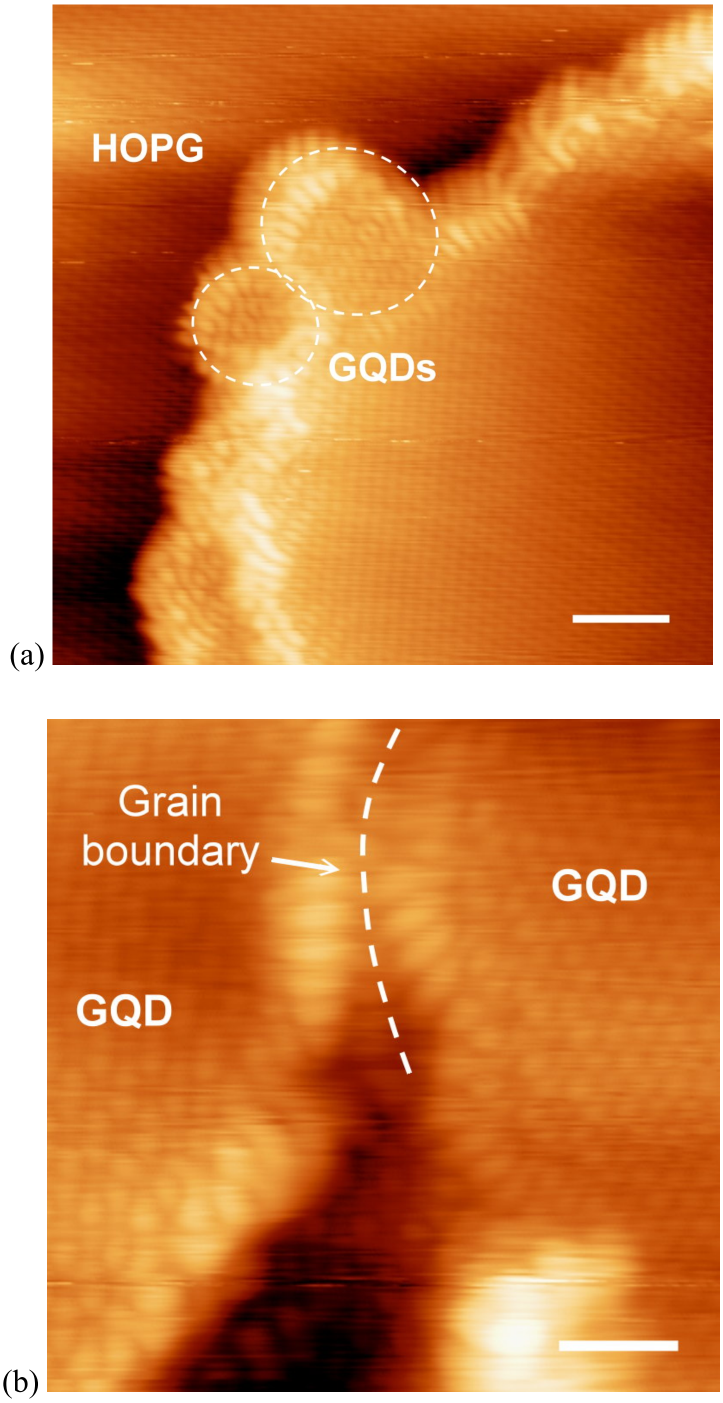

Supplementary Fig. 11. STM images of P-GQDs growth on HOPG. The scale bars are 2 nm in (a) and 1 nm in (b).

Supplementary Fig. 12

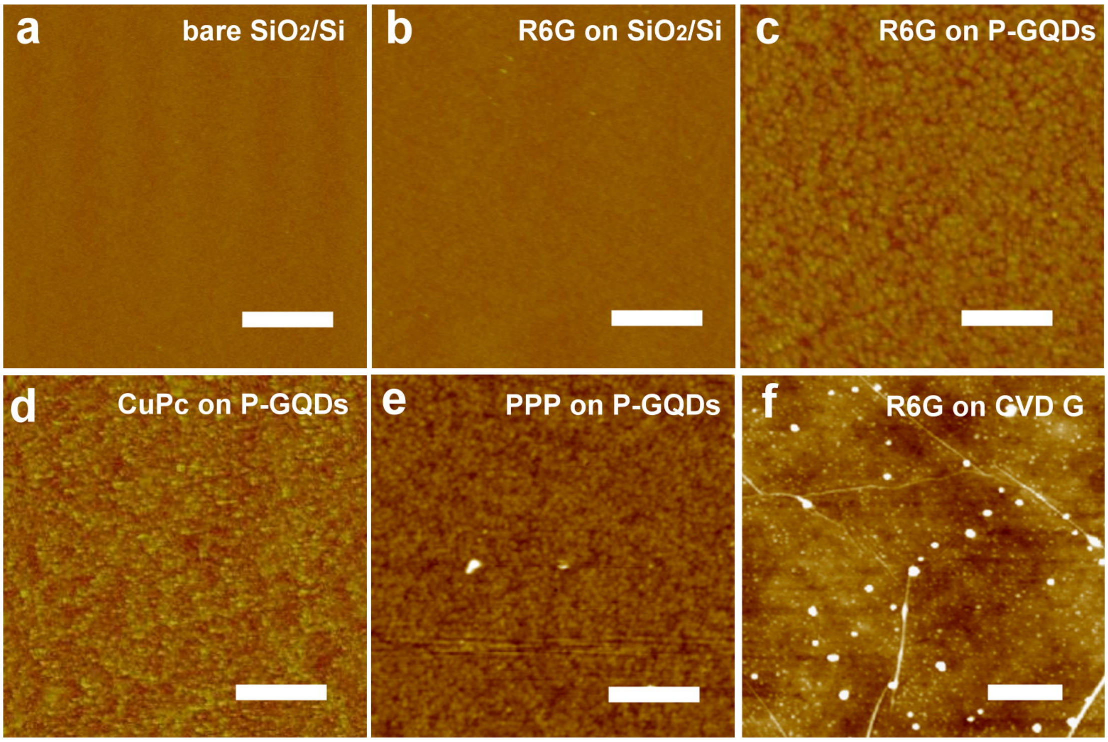

Supplementary Fig. 12. AFM images of (a) the bare SiO_2/Si, (b) SiO_2/Si with thermally evaporated 0.2 nm R6G, (c) P-GQDs with thermally evaporated 0.2 nm R6G, (d) P-GQDs with thermally evaporated 0.2 nm CuPc, (e) P-GQDs with thermally evaporated 0.2 nm PPP, and (f) CVD graphene with thermally evaporated 0.2 nm R6G. The impurties on CVD graphene are introduced by the post-growth transfer process. The scale bars are 500 nm.

Supplementary Fig. 13

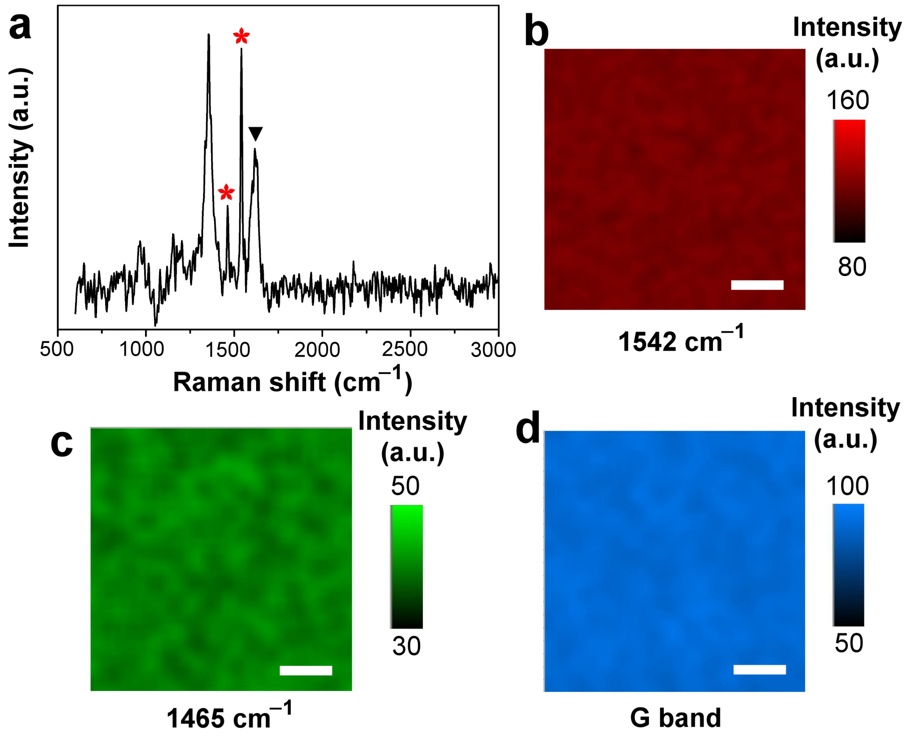

Supplementary Fig. 13. (a) Raman spectrum of thermally evaporated CuPc on P-GQDs. Black arrow indicates the G band of graphene, and red stars indicate the peaks from CuPc. (b-d) Raman mapping of the intensity of the characteristic peaks of CuPc and the G band of P-GQDs. The scale bars are 10 μm.

Supplementary Fig. 14

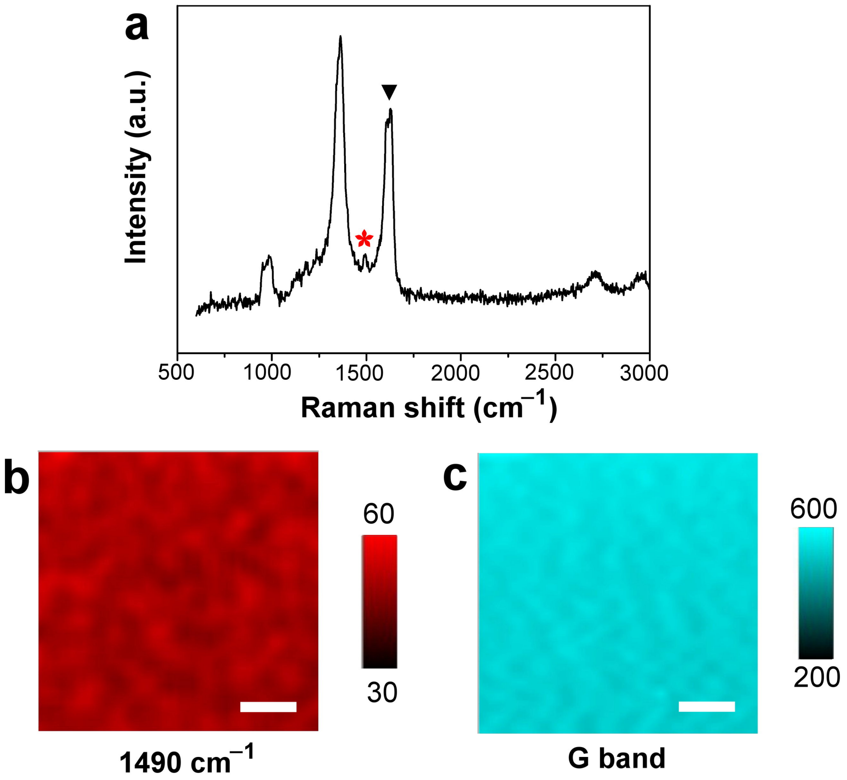

Supplementary Fig. 14. (a) Raman spectrum of thermally evaporated PPP on P-GQDs. Black arrow indicates the G band of graphene, and red star indicates the peaks from PPP. (b, c) Raman mapping of the intensity of the characteristic peak of PPP and the G band of P-GQDs. The scale bars are 10 μm.

Supplementary Fig. 15

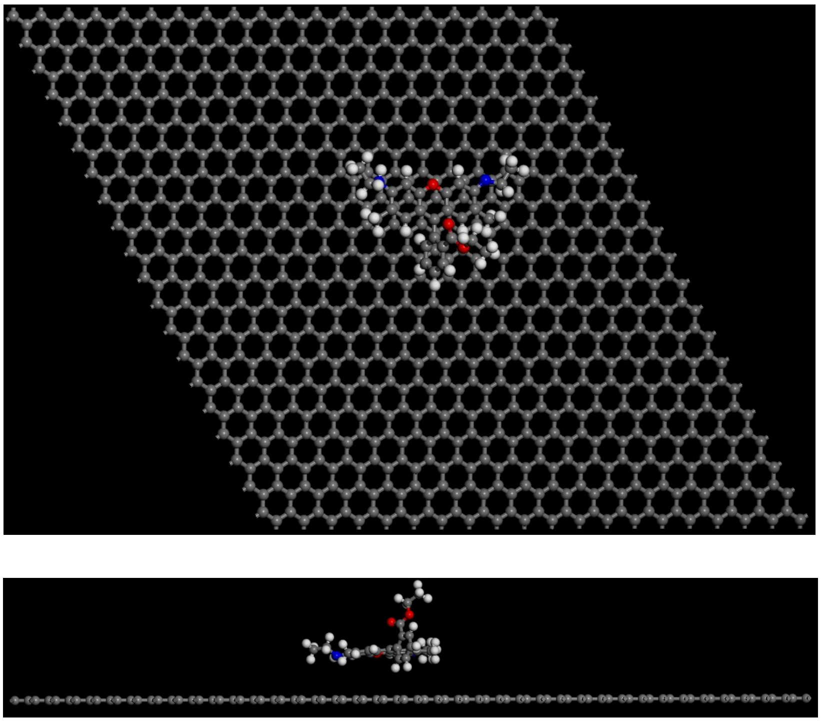

(a)

(b)

Supplementary Fig. 15. The model of the R6G/graphene model, which was used in the *ab initio* DFT calculation. (a) Top and (b) side views of the model. The details of the model are shown in the Methods.

Supplementary Fig. 16

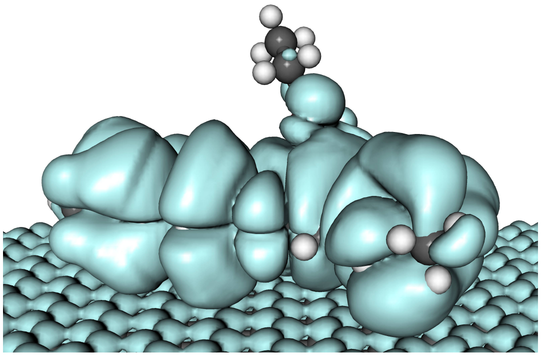

(a)

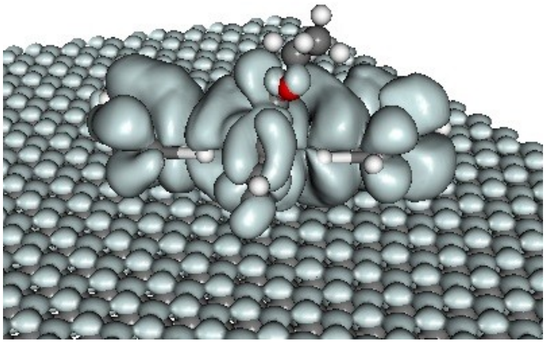

(b)

Supplementary Fig. 16. The molecular orbital (at the HOMO level of R6G) densities obtained from DFT for the R6G/graphene system. (a) Front and (b) back views of the molecular orbital.

Supplementary Fig. 17

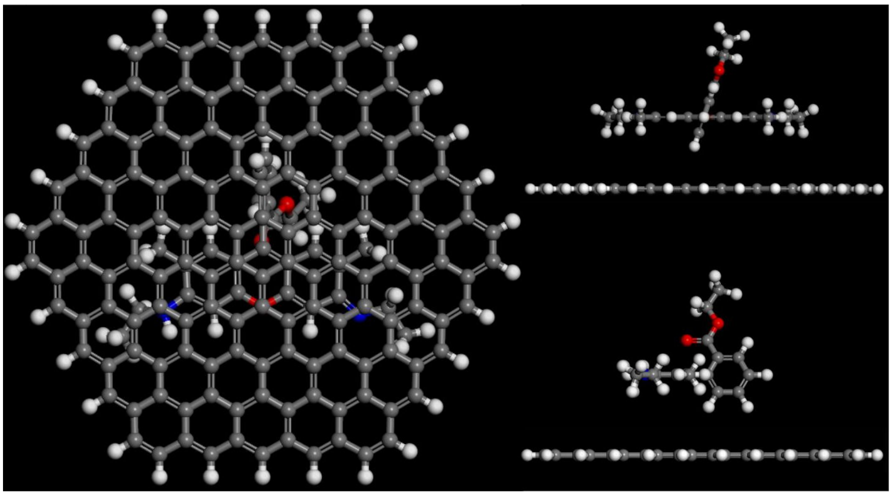

Supplementary Fig. 17. Top and two side views of the R6G/GQD (2.2 nm) model, which was used in the *ab initio* DFT calculation. The details of the model are shown in the Methods.

Supplementary Fig. 18

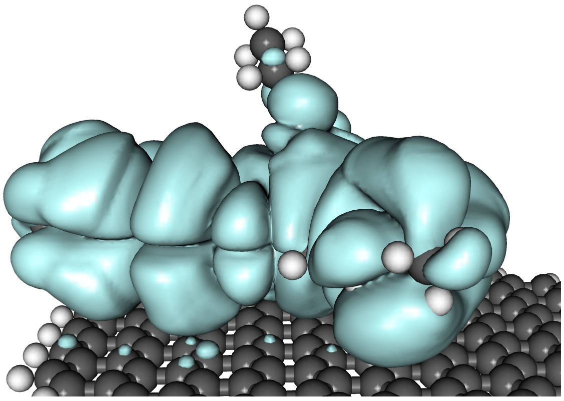

(a)

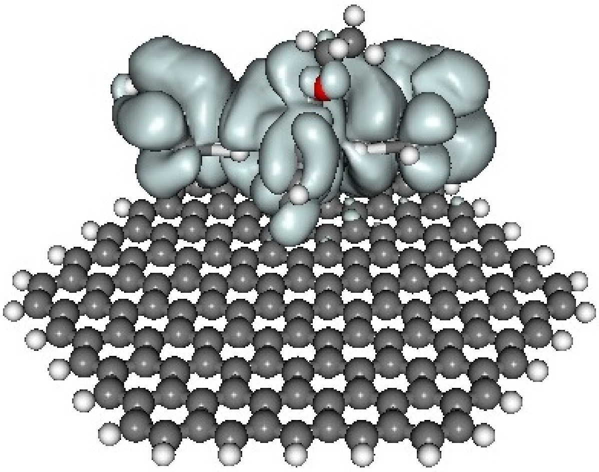

(b)

Supplementary Fig. 18. The molecular orbital (at the HOMO level of R6G) densities obtained from DFT for the R6G/GQD (2.2 nm) system. (a) Front and (b) back views of the molecular orbital.

Supplementary Fig. 19

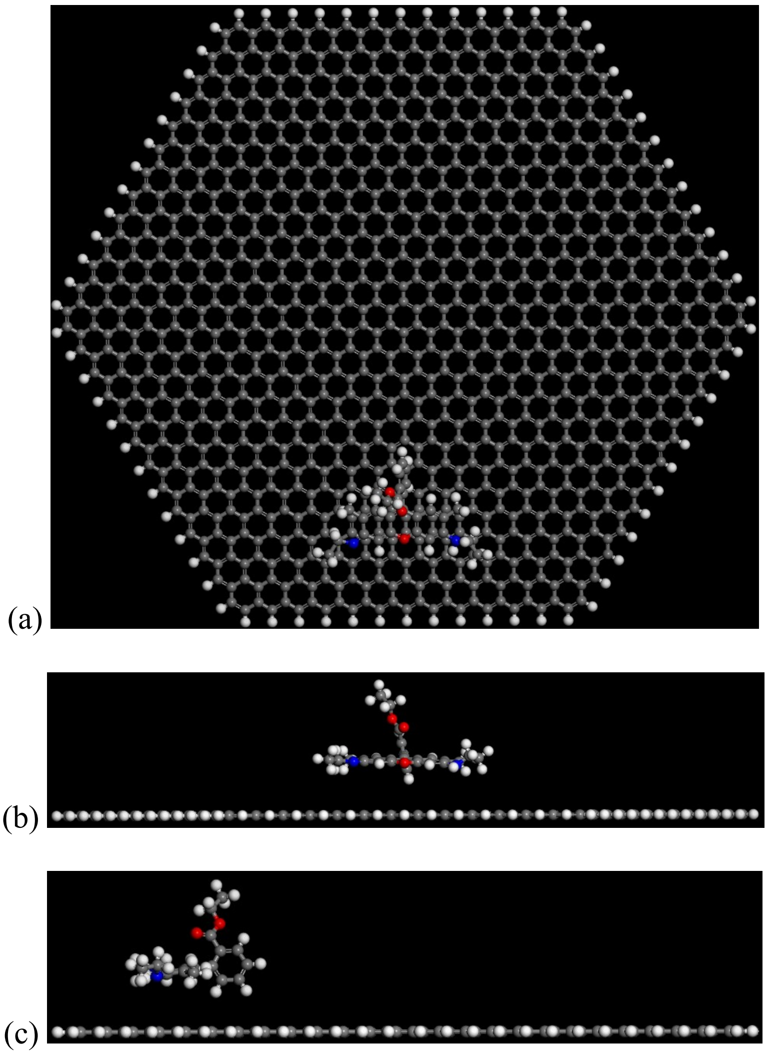

(a) (b) (c)

Supplementary Fig. 19. The R6G/GQD (6.2 nm) model, which was used in the *ab initio* DFT calculation. (a) Top and (b,c) two side views of the model. The details of the model are shown in the Methods.

Supplementary Fig. 20

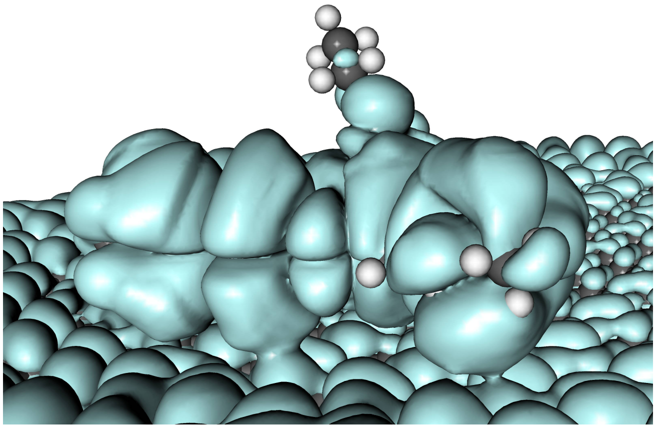

(a)

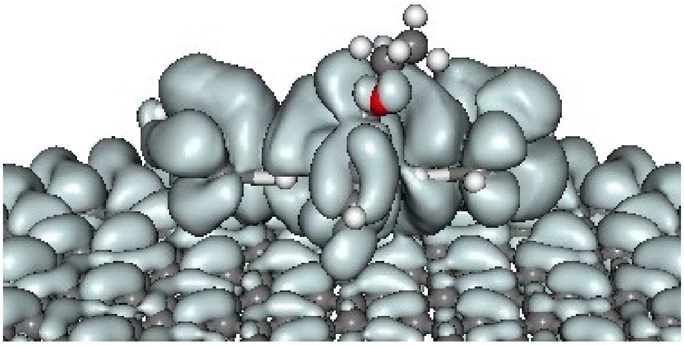

(b)

Supplementary Fig. 20. The molecular orbital (at the HOMO level of R6G) densities obtained from DFT for the R6G/GQD (6.2 nm) system. (a) Front and (b) back views of the molecular orbital.

Supplementary Fig. 21

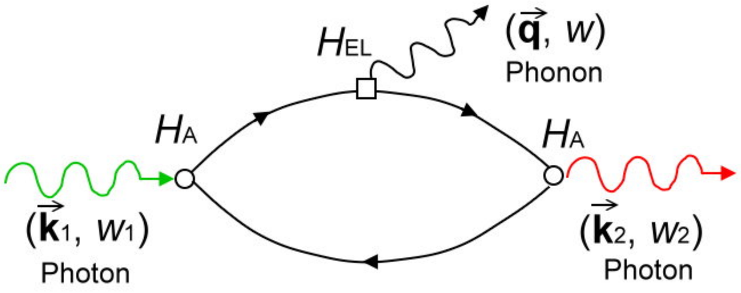

Supplementary Fig. 21. Feynman diagram for one-phonon (Stokes) Raman scattering[20]. $\mathbf{k}_1$, w_1 and $\mathbf{k}_2$, w_2 are the incident and scattered photon wavevectors and freqencies. H_A and H_{EL} are the interactions of the electrons with the radiation and lattice. $\mathbf{q}$ and w are the wavevector and freuency of the elementary excitation created.

Supplementary Fig. 22

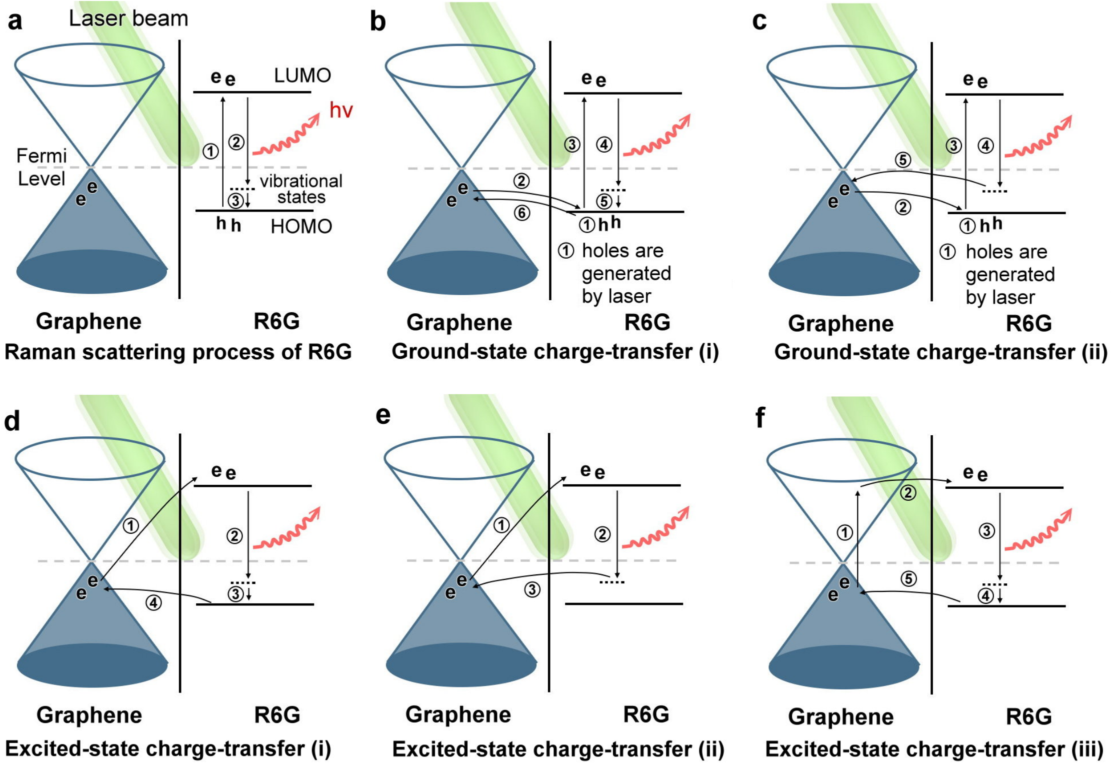

Supplementary Fig. 22. Possible charge transport processes in the Raman scattering of the R6G. (a) The Raman scattering process of the R6G. (b,c) The possible electron process in ground-state charge transfer. (d-f) The possible electron process in excited-state charge transfer.

Supplementary Fig. 23

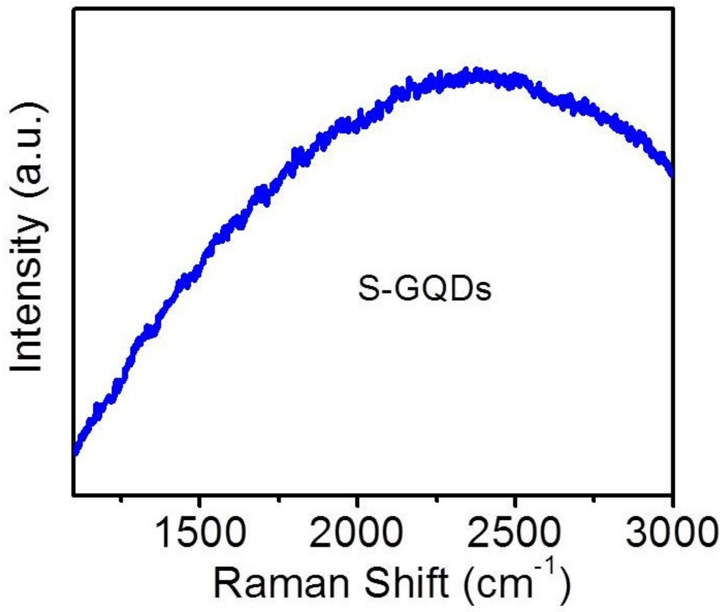

(a)

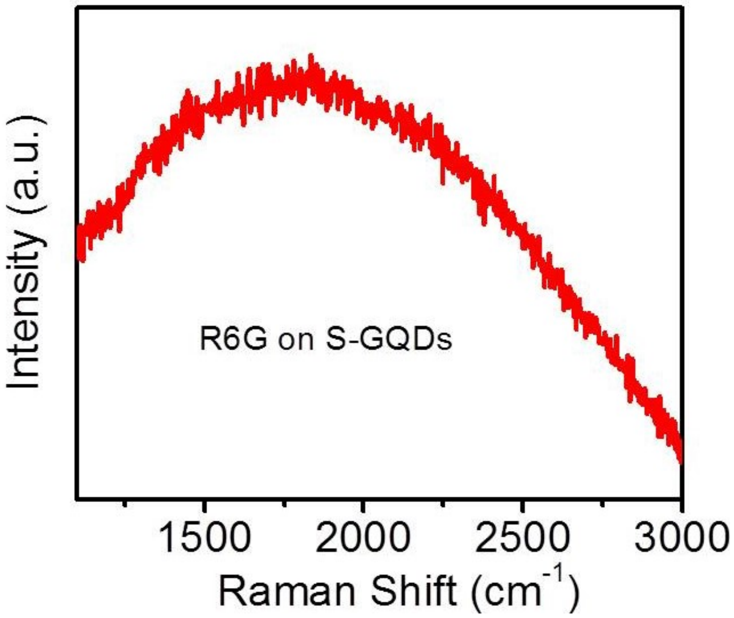

(b)

Supplementary Fig. 23. Raman spectra of S-GQDs produced by polymerization and carbonization reactions (a) before and (b) after depositing R6G (10^{-5} mol L^{-1}) molecules.

Supplementary Fig. 24

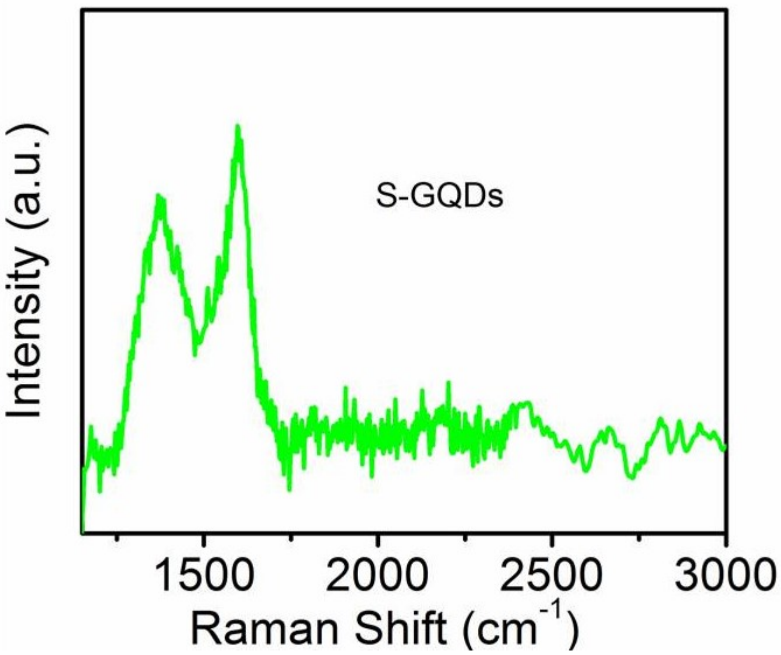

(a)

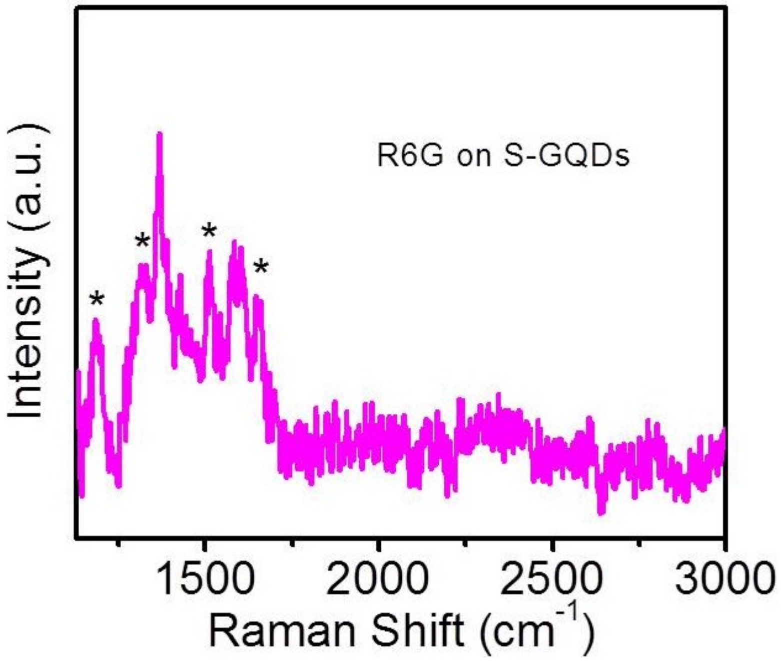

(b)

Supplementary Fig. 24. Raman spectra of S-GQDs produced by cutting the graphene oxide sheets using hydrothermal reactions (a) before and (b) after depositing R6G (10^{-5} mol L^{-1}) molecules.

Supplementary Fig. 25

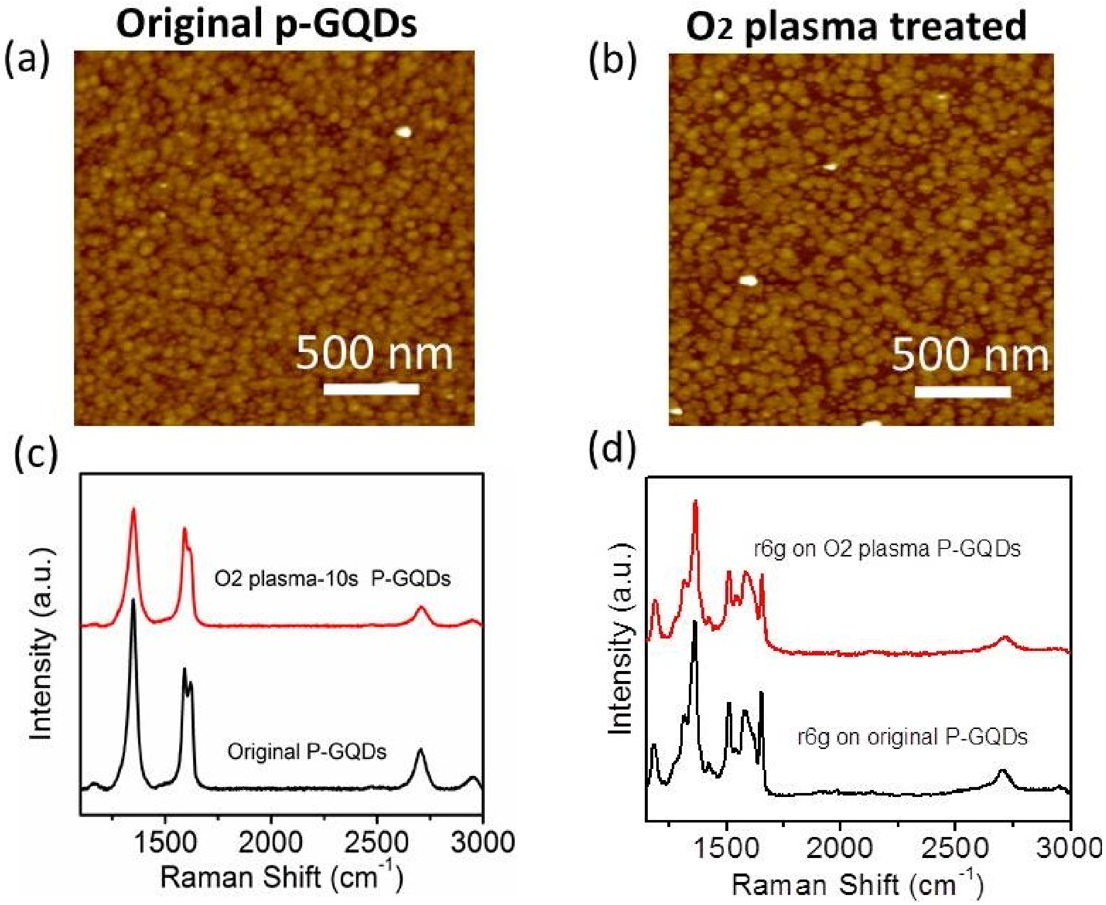

Supplementary Fig. 25. (a, b) AFM images of P-GQDs (a) before and (b) after O_2 plasma treatment for 10 s. (c) Raman spectra of P-GQDs before and after O_2 plasma treatment. (d) Raman spectra of R6G on original P-GQDs and O_2 plasma treated P-GQDs.

Supplementary Fig. 26

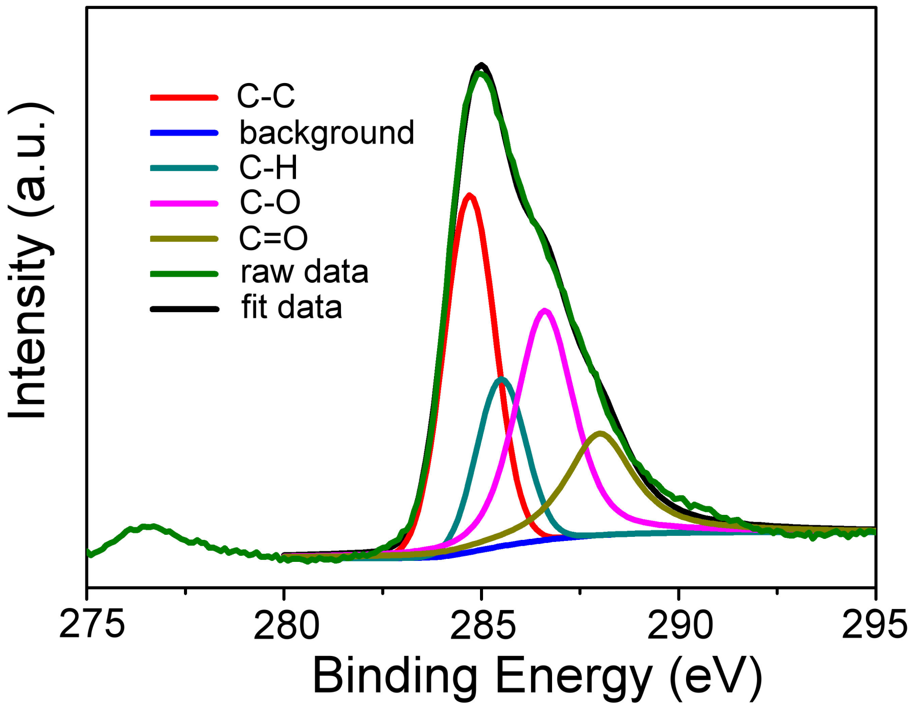

Supplementary Fig. 26. XPS C1s spectrum of the P-GQDs after O_2 plasma treatment for 10 s.

Supplementary Fig. 27

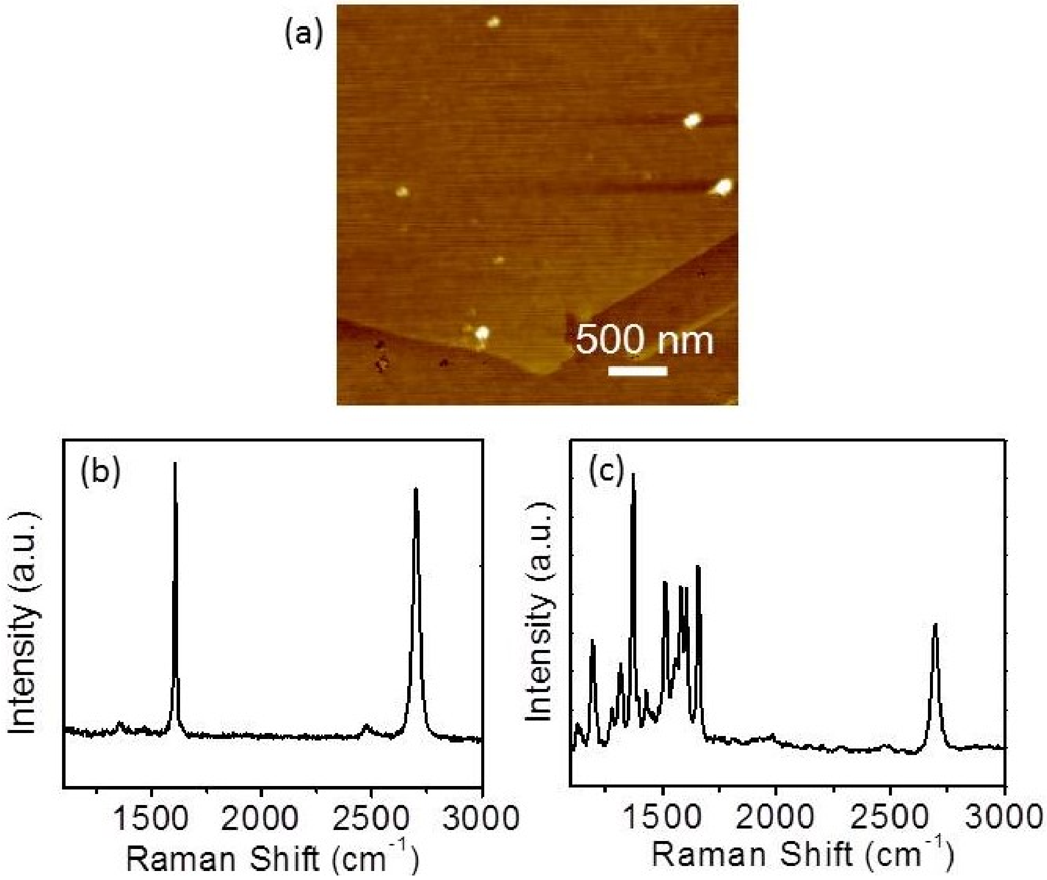

Supplementary Fig. 27. (a) AFM images of exfoliated graphene. (b, c) Raman spectra of the exfoliated graphene before and after depositing R6G molecules.

Supplementary Fig. 28

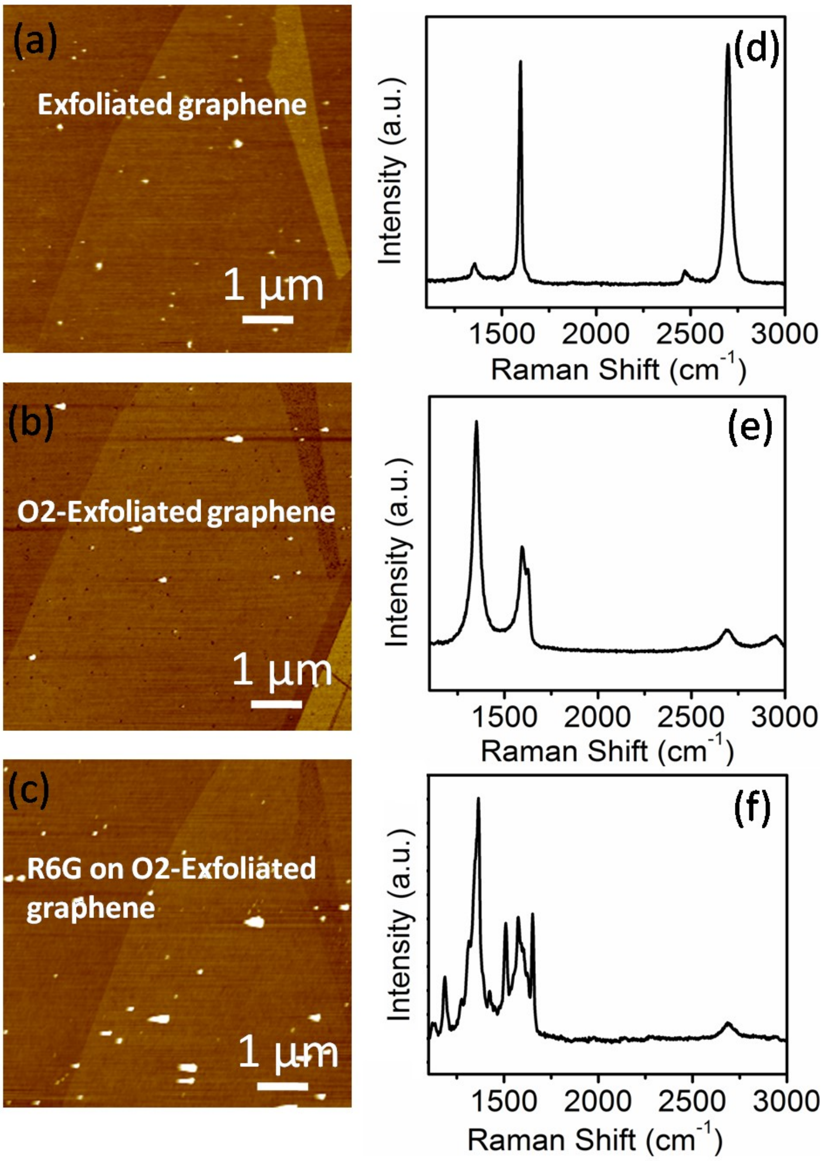

Supplementary Fig. 28. (a-c) AFM images of (a) the original exfoliated graphene, exfoliated graphene (b) after O_2 plasma treatment and (c) after depositing R6G on it. (d-f) Raman spectra of (d) the original exfoliated graphene, (e) exfoliated graphene after O_2 plasma treatment and (f) after depositing R6G molecules on it.

Supplementary Fig. 29

(a)

(b)

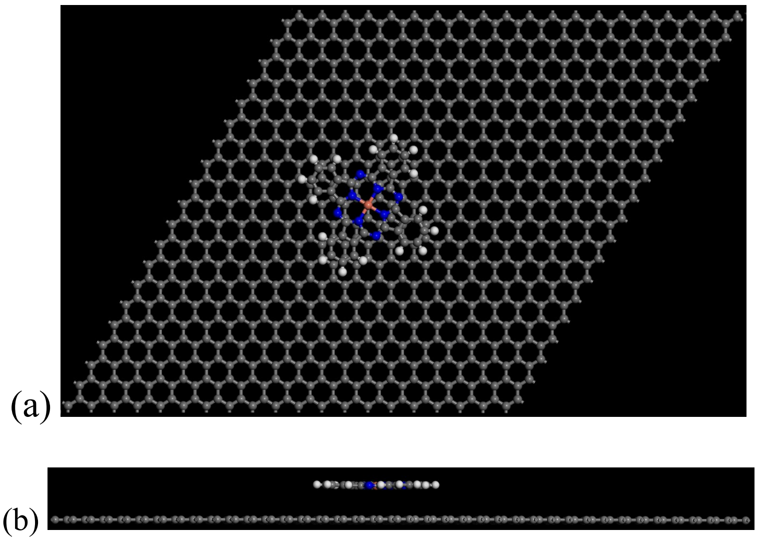

Supplementary Fig. 29. The model of the CuPc/graphene model. (a) Top and (b) side views of the model.

Supplementary Fig. 30

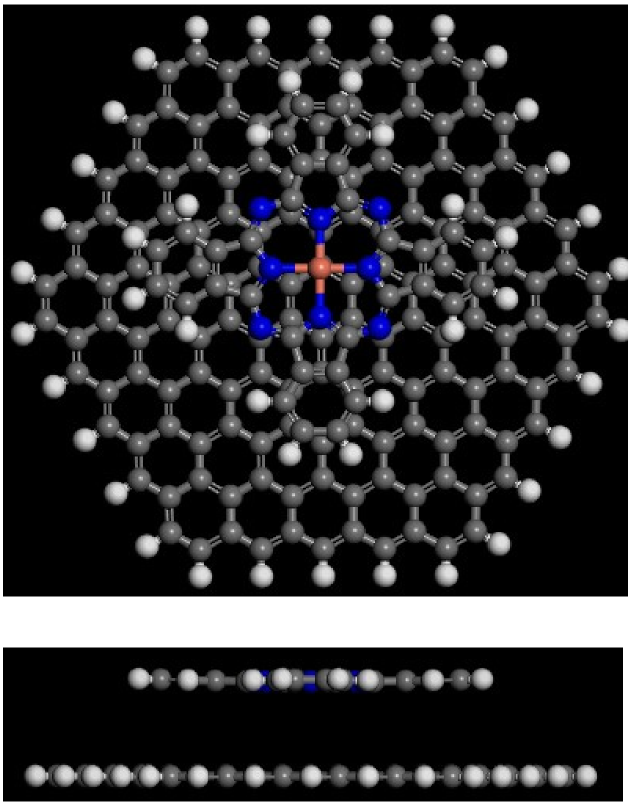

(a) (b)

Supplementary Fig. 30. The model of the CuPc/GQD (2.2 nm). (a) Top and (b) side views of the model.

Supplementary Fig. 31

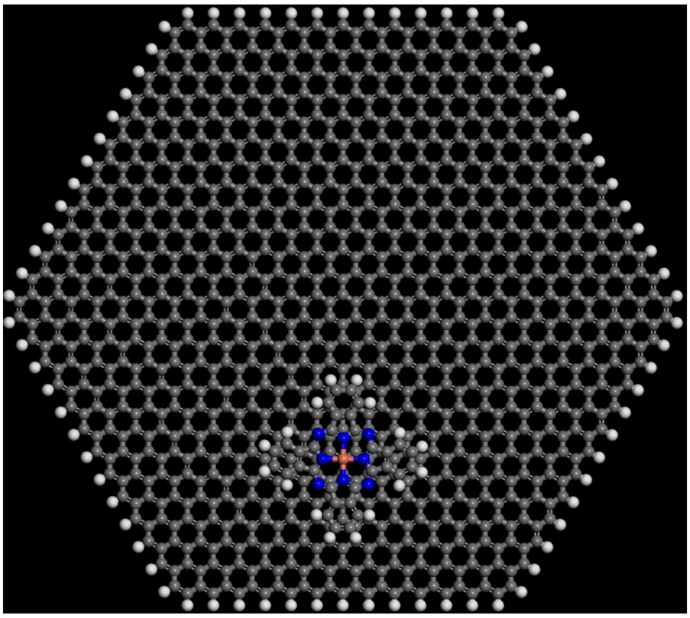

(a)

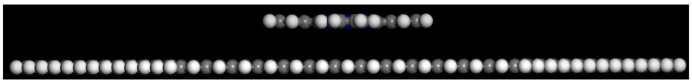

(b)

Supplementary Fig. 31. The model of the CuPc/GQD (6.2 nm) model. (a) Top and (b) side views of the model.

Supplementary Fig. 32

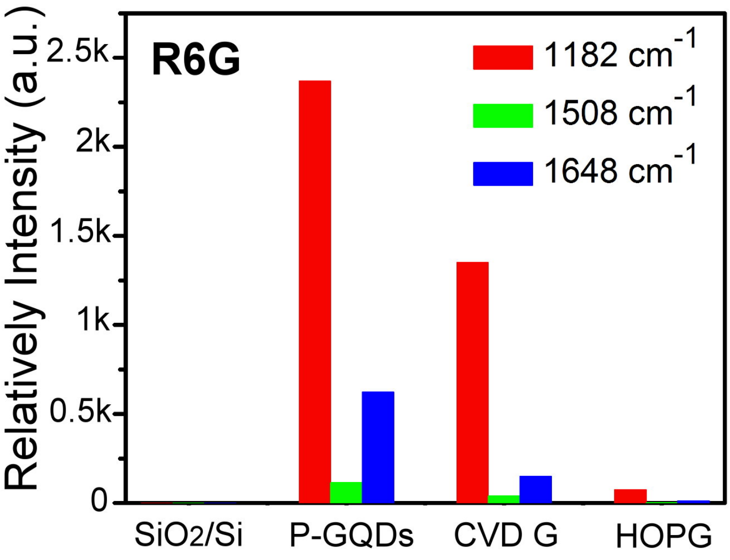

Supplementary Fig. 32. Relative intensity of the characteristic peaks of R6G (10^{-5} mol L^{-1}) on different substrates, normalized to the signals on SiO_2/Si.

Supplementary Fig. 33

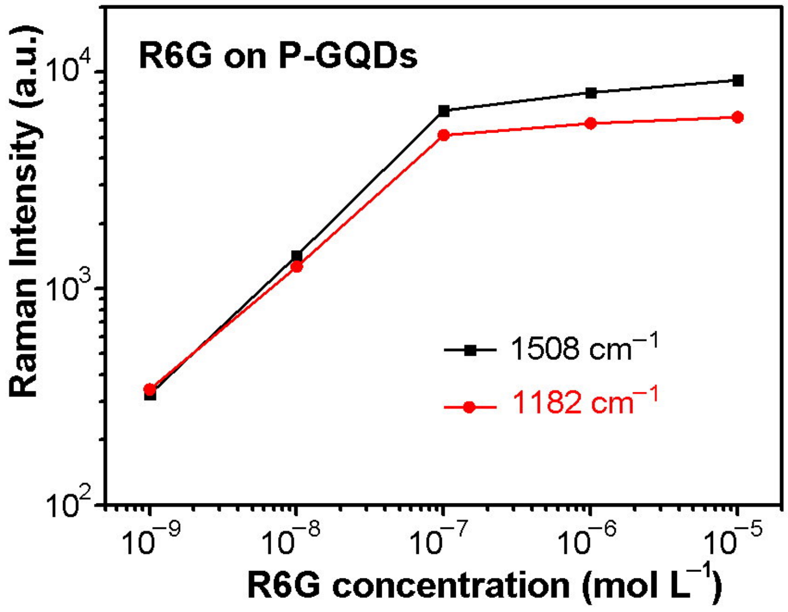

Supplementary Fig. 33. Raman intensity of the characteristic peaks (1508 cm^{-1} and 1182 cm^{-1}) of R6G on P-GQDs. R6G molecules were deposited by dropping 10^{-9}, 10^{-8}, 10^{-7}, 10^{-6}, 10^{-5} mol L^{-1} R6G solution on P-GQDs.

Supplementary References

1. Wei, D. C., Lu, Y. H., Han, C., Niu, T. C., Chen, W., Wee, A. T. S. Critical crystal growth of graphene on dielectric substrates at low temperature for electronic devices. *Angew.Chem. Int. Ed.* **52,** 14121–14126 (2013).

2. Liu, D. H., Yang, W., Zhang, L. C., Zhang, J., Meng, J. L., Yang, R., Zhang, G. Y., Shi, D. X. Two-step growth of graphene with separate controlling nucleation and edge growth directly on SiO_2 substrate. *Carbon* **72,** 387–392 (2014) .

3. Ryu, H. J., Kim, S. H., Hong, S. H. Effect of deposition pressure on bonding nature in hydrogenated amorphous carbon films processed by electron cyclotron resonance plasma enhanced chemical vapor deposition. *Mater. Sci. Eng. A* **277,** 57–63 (2000).

4. Herrebout, D., Bogaerts, A., Yan, M., Gijbels, R., Goedheer, W., Dekempeneer, E. One-dimensional fluid model for an rf methane plasma of interest in deposition of diamond-like carbon layers. *J. Appl. Phys.* **90,** 570–579 (2001).

5. Xie, L. M., Jiao, L. Y., Dai, H. J. Selective etching of graphene edges by hydrogen plasma. *J. Am. Chem. Soc.* **132,** 14751−14753 (2010) .

6. Liu, Y., Tian, Y., Tian, Y., Wang, Y., Yang, W. Carbon-dot-based nanosensors for the detection of intracellular redox state. *Adv. Mater.* **27,** 7156–7160 (2015).

7. Zhu, S., Meng, Q., Wang, L., Zhang, J., Song, Y. B., Jin, H., Zhang, K., Sun, H., Yang, B. Highly photoluminescent carbon dots for multicolor patterning, sensors, and bio-imaging. *Angew. Chem. Int. Ed.* **52,** 3953–3957 (2013).

8. Pan, D., Zhang, J., Li, Z., Wu, M. Hydrothermal route for cutting graphene sheets into blue-luminescent graphene quantum dots. *Adv. Mater.* **22,** 734–738 (2010).

9. Lippitsch, M. E. Ground-state charge transfer as a mechanism for surface-enhanced Raman scattering. *Phys. Rev. B* **29,** 3101 (1984).

10. Adrian, F. J. Charge transfer effects in surface - enhanced Raman scattering. *J. Chem. Phys.* **77,** 5302–5314 (1982).

11. Xu, H., Xie, L. M., Zhang, H. L. and Zhang, J. Effect of graphene Fermi level on the Raman scattering intensity of molecules on graphene. *ACS Nano* **5,** 5338−5344 (2011).

12. Xue, T., Yu, S. S., Zhang, X. M., Zhang, X. Z., Wang, L., Bao, Q. L., Chen, C., Zheng, W. T., Cui, X. Q. R6G molecule induced modulation of the optical properties of reduced graphene oxide nanosheets for use in ultrasensitive SPR sensing. Sci. Rep. 6, 21254 (2016).

13. Huang, C., Kim, M., Wong, B. M., Safron, N. S., Arnold, M. S., Gopalan, P. Raman Enhancement of a Dipolar Molecule on Graphene. J. Phys. Chem. C 118, 2077–2084 (2014).

14. Deng, W. Q. and Goddard III, W. A. Predictions of hole mobilities in oligoacene organic semiconductors from quantum mechanical calculations. *J. Phys. Chem. B* **108,** 8614–8621 (2004).

15. Zhang, Y., Qiao, J., Gao, S., Hu, F., He, D., Wu, B., Yang, Z., Xu, B., Li, Y., Shi, Y., Ji, W., Wang, P., Wang, X., Xiao, M., Xu, H., Xu, J.-B. and Wang, X. R. Probing carrier transport and Sstructure-property relationship of highly ordered organic semiconductors at the two-dimensional limit. *Phys. Rev. Letts*. **116,** 016602 (2016).

16. Tao, Y. S., Mao, H. Y., He, P. M. Electronic and structural properties at the interface between CuPc and graphene. *J. Appl. Phys.* **117,** 013701 (2015).

17. Jomphoak, A., Maezono, R., Onjun, T. Density functional theory of graphene/Cu phthalocyanine composite material. *Surface & Coating Technology,* **306A,** 236−239 (2016).

18. Bialek, B., Kim, I. G., Lee, J. I. Electronic structure of copper phthalocyanine monolayer: a first-principles study. *Thin Solid Films* **436,** 107–114 (2003).

19. Aristov, V. Y., Molodtsova, O. V., Maslyuk, V., Vyalikh, D. V., Zhilin, V. M., Ossipyan, Y., Bredowe, T., Mertig, I., Knupfer, M. Electronic structure of pristine CuPc: Experiment and calculations. *Appl. Surf. Sci.* 254, 20−25 (2007).

20. M. Cardona, "Light Scattering in Solids I", Springer-Verlag, Berlin, 1 (1984).
